# Genomic Organization, Phylogenetic Comparison and Differential Expression of the SBP-Box Family Genes in Grape

**DOI:** 10.1371/journal.pone.0059358

**Published:** 2013-03-19

**Authors:** Hongmin Hou, Jun Li, Min Gao, Stacy D. Singer, Hao Wang, Linyong Mao, Zhangjun Fei, Xiping Wang

**Affiliations:** 1 State Key Laboratory of Crop Stress Biology in Arid Areas, College of Horticulture, Northwest A&F University, Yangling, Shaanxi, China; 2 Key Laboratory of Horticultural Plant Biology and Germplasm Innovation in Northwest China, Ministry of Agriculture, Northwest A&F University, Yangling, Shaanxi, China; 3 Department of Agricultural, Food and Nutritional Science, University of Alberta, Edmonton, Alberta, Canada; 4 Boyce Thompson Institute for Plant Research, Cornell University, Ithaca, New York, United States of America; 5 USDA Robert W. Holley Center for Agriculture and Health, Ithaca, New York, United States of America; Cankiri Karatekin University, Turkey

## Abstract

**Background:**

The SBP-box gene family is specific to plants and encodes a class of zinc finger-containing transcription factors with a broad range of functions. Although SBP-box genes have been identified in numerous plants including green algae, moss, silver birch, snapdragon, *Arabidopsis*, rice and maize, there is little information concerning SBP-box genes, or the corresponding *miR156/157*, function in grapevine.

**Methodology/Principal Findings:**

Eighteen SBP-box gene family members were identified in *Vitis vinifera*, twelve of which bore sequences that were complementary to *miRNA156/157*. Phylogenetic reconstruction demonstrated that plant SBP-domain proteins could be classified into seven subgroups, with the *V. vinifera* SBP-domain proteins being more closely related to SBP-domain proteins from dicotyledonous angiosperms than those from monocotyledonous angiosperms. In addition, synteny analysis between grape and *Arabidopsis* demonstrated that homologs of several grape SBP genes were found in corresponding syntenic blocks of *Arabidopsis*. Expression analysis of the grape SBP-box genes in various organs and at different stages of fruit development in *V. quinquangularis* ‘Shang-24’ revealed distinct spatiotemporal patterns. While the majority of the grape SBP-box genes lacking a *miR156/157* target site were expressed ubiquitously and constitutively, most genes bearing a *miR156/157* target site exhibited distinct expression patterns, possibly due to the inhibitory role of the microRNA. Furthermore, microarray data mining and quantitative real-time RT-PCR analysis identified several grape SBP-box genes that are potentially involved in the defense against biotic and abiotic stresses.

**Conclusion:**

The results presented here provide a further understanding of SBP-box gene function in plants, and yields additional insights into the mechanism of stress management in grape, which may have important implications for the future success of this crop.

## Introduction

Transcription factors, which are proteins that bind DNA in a sequence-specific manner and regulate gene expression by activating or repressing the transcription of downstream target genes, are found in virtually all living organisms and play an essential role in regulatory networks of many important developmental processes. SQUAMOSA PROMOTER BINDING PROTEIN (SBP)-box genes encode a family of plant-specific transcription factors [1], [2] that contain a highly conserved DNA-binding domain termed the SBP domain. This domain is an assembly of approximately 76 amino acid residues that are involved in both DNA-binding and nuclear localization, and features two zinc-binding sites [2], [3]. SBP-box genes were first identified in *Antirrhinum majus* (*AmSBP1* and *AmSBP2*) based on the ability of their encoded proteins to interact with the promoter region of the floral meristem identity gene *SQUAMOSA* [2].

In *Arabidopsis*, the first SBP-box gene identified was *SQUAMOSA PROMOTER BINDING PROTEIN-LIKE3* (*SPL3*), which was found to be involved in the floral transition. Similar to the AmSBP1 and AmSBP2 proteins from *A. majus*, AtSPL3 recognizes a conserved motif in the promoter region of the *Arabidopsis* floral meristem identity gene *APETALA1* (*AP1*), which is an ortholog of *SQUAMOSA* [4]. To date, sixteen SBP-box genes have been identified in the *Arabidopsis* genome [1], [5], of which some have been shown to function in regulation of plant development, ranging from sporogenesis [6], shoot development [7], flowering [8], plastochron formation [9], vegetative and reproductive phase transitions [10], [11], leaf development [12], fertility [13], to plant hormone signaling [14] and copper homeostasis [15].

In recent years, knowledge concerning the functions of SBP-box genes in plant species other than *Arabidopsis* has also begun to accumulate and highlights the diverse roles of these proteins in plant development. For example, a novel SBP-box gene, *BpSPL1*, from silver birch (*Betula pendula*) was shown to be capable of binding a *cis*-element of *BpMADS5*, which is a close homolog of the *Arabidopsis FRUITFULL* gene, and indicated a role for birch SBP-box genes in the regulation of flower development [16]. Similarly, *AmSBP1* from *A. majus* has recently been implicated in flowering initiation through the activation of meristem identity genes [17]. In contrast, a tomato SBP-box gene has been found to be a critical factor in fruit ripening [18], while the maize *tasselsheath4* (*tsh4*) SBP-box gene has been shown to regulate bract development and to be a necessity in branch meristem initiation and maintenance [19]. Conversely, in the unicellular alga, *Chlamydomonas reinhardtii*, an SBP-domain protein has been determined to regulate nutritional copper signaling [20]. Finally, of the 19 putative SBP*-*like genes identified and characterized in the rice (*Oryza sativa*) genome, more than half were shown to be highly expressed in young panicles, suggesting their putative roles in these organs [21].

In recent years, an increasing number of microRNAs (miRNAs) have been found to play a crucial role in the regulation of gene function in plants, and these numbers are still accumulating at present. MiRNAs are small RNA molecules (20–24 nucleotides in length) that can cause the degradation of mRNAs or repress translation by binding to the transcripts of their target genes, of which approximately half encode transcription factors [22], and forming an RNA-induced silencing complex. As a gene family encoding transcription factors, more than half of the SBP-box genes identified to date have been found to be targeted by *miR156*/*157*. In rice, for instance, 11 of the 19 SBP-box genes have been revealed to be putative targets of *OsmiR156* [21], while 10 of 15 SBP-box gene family members in tomato were found to carry putative *miR156*/*157*-response elements [23].

Unfortunately, despite an increasing body of physiological and biochemical data, the biological role of the SBP-box transcription factor-encoding gene family remains elusive. Although grapevine (*V. vinifera*) is one of the most important perennial fruit crops worldwide, there is little information concerning SBP-box gene, or the corresponding *miR156/157*, function in this species [24]. In the present study, we first systematically performed a genome-wide identification of SBP-box genes in the *V. vinifera* genome and then conducted further gene classification through an examination of exon–intron structure, gene phylogeny and synteny analysis. Since various types of stresses can cause significant losses in grape yield and reduce berry quality, we also endeavored to investigate expression patterns of grape SBP-box genes under various abiotic and biotic stresses, as well as in response to particular phytohormone treatments. This was carried out by both mining publicly available microarray datasets and through the examination of transcription levels in various grape organs and at different stages of fruit development. The results obtained from this study provide a foundation for additional evolutionary and functional characterization of SBP-box genes in plants, and further our understanding of stress management strategies utilized in grape.

## Results

### Identification of Grape SBP-box Genes in the *V. vinifera* Genome

Previously, a subset of SBP-box genes were identified in grape; however, the results were incomplete and included a number of genes that lacked a complete SBP-box [24].Therefore, in an effort to identify the entire collection of putative SBP-box genes in the grape genome, we searched the GenBank non-redundant protein database, as well as the Grape Genome Database (12X) (http://www.genoscope.cns.fr), with a profile Hidden Markov Model (pHMM) of the SBP domain(PF03110). Following the removal of redundant sequences, we identified 20 putative protein sequences. However, two of these sequences (GSVIVT01020050001 and GSVIVT01020051001) contained no zinc-finger motif and were thus excluded from further analysis. Therefore, we determined that at least 18 putative SBP-box genes were present in the *V. vinifera* genome, which we termed *VvSBP1* to *VvSBP18* (to maintain simplicity we also used this nomenclature when referring to orthologs in other grape species) based on their chromosomal order. In this study, to determine or validate the predicted exon–intron structures,the complete open reading frames of all the grape SBP genes were isolated from cDNA of *V. quinquagularis* ‘Shang-24’ with gene-specific primers (Table S1, Fig. S1). Amplified products were sequenced and corroborated the expression of all predicted grape SBP genes. Moreover, the sequences of SBP genes amplified were consistent with those published in GenBank.

**Figure 1:**
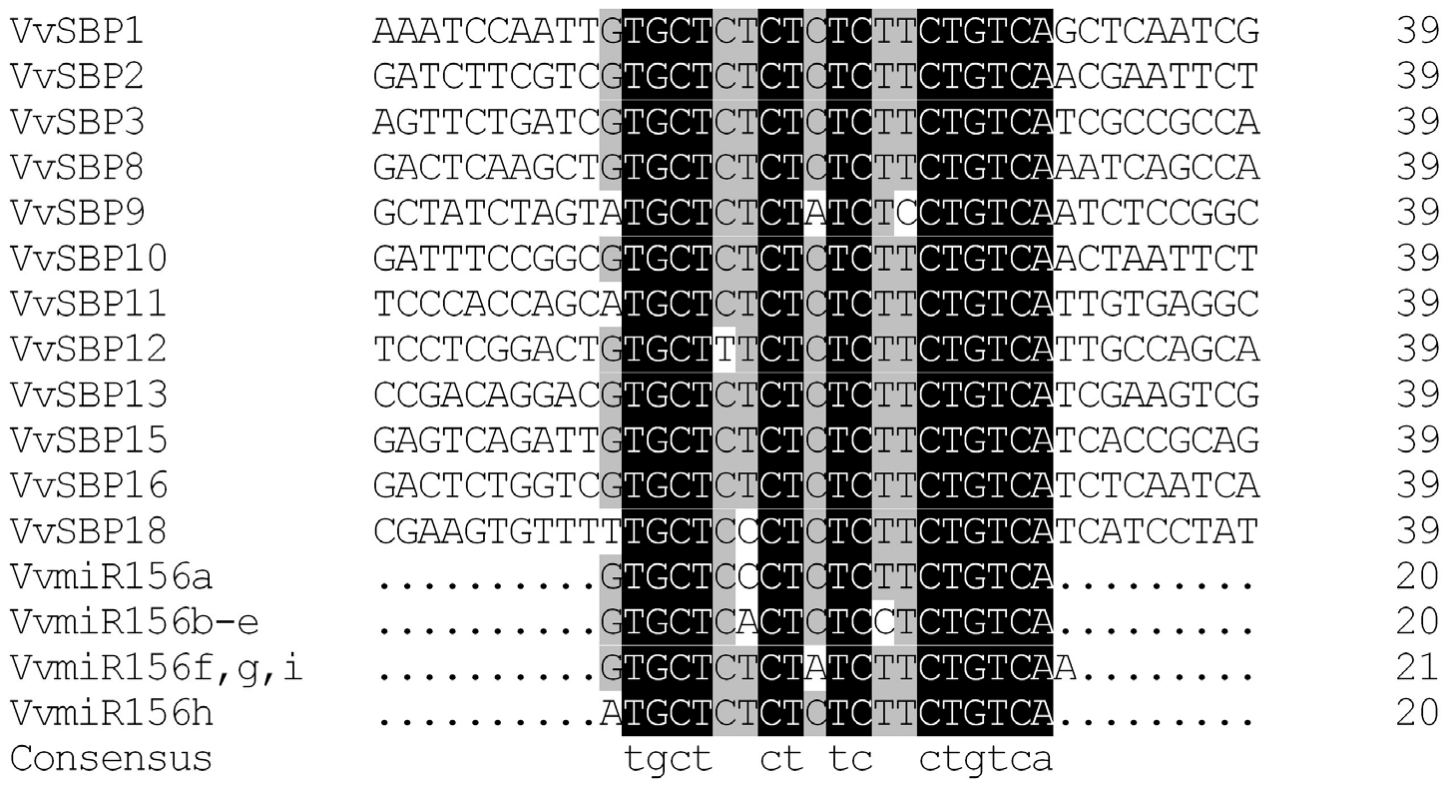
Alignment of *miR156/157* complementary sequences within grape SBP genes. Complementary sequences are within coding regions, with the exception of *VvSBP9*, *VvSBP11* and *VvSBP18* where they are located within the 3′ UTR. Reverse complement sequences of the mature *VvmiR156* genes are shown below the alignment for comparison.

In addition, in accordance with findings in other species, we also found that 12 of the 18 grape SBP genes identified in this study contained sequences that were complementary to *miR156/157*, with a maximum of one to three mismatches to the mature *VvmiR156/157* sequences (Fig. 1).

### Phylogenetic Analysis of SBP-domain Family Genes

**Figure 2:**
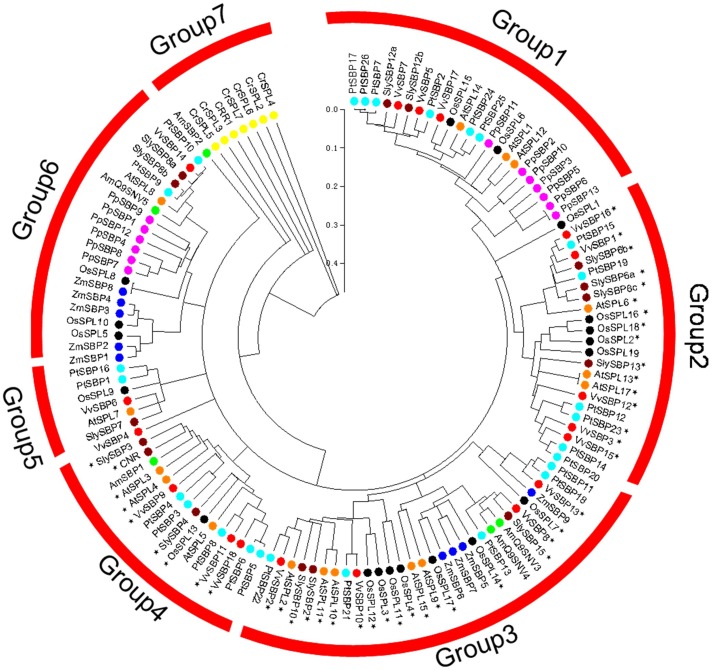
Unrooted phylogenetic tree of the SBP-box family genes based on amino acid sequences of SBP domains. Red bars denote different groups (or subgroups) of SBP domains. Circles of different colors represent SBP domain proteins from different species. SBP-box genes from *Arabidopsis*, rice, tomato, and grape that contain complementary sequences for *miR156/157* are marked with an asterisk. SBP domain sequences of all genes utilized in this analysis, as well as the accession numbers and data sources of genes from plants are listed in Tables S2 and S3.

In an effort to gain further insight into the evolutionary relationship between SBP-box genes in various species of plants, we selected 128 SBP-box genes from nine species, including seven from the green alga *C. reinhardtii*, fourteen from the moss *P. patens*, eighty from dicotyledonous angiosperms (*Arabidopsis, Vitis* spp., *Solanum* spp., *A. majus* and poplar), as well as twenty-seven from monocotyledonous angiosperms (rice and maize) (Table S2), and constructed a phylogenetic tree based on the encoded amino acid sequences of their highly conserved SBP domains (76 aa) using the neighbor-joining algorithm (Table S3). The resulting unrooted phylogenetic tree suggested that the plant SBP-domain family is evolutionarily diverse (Fig. 2), with the 128 plant SBP sequences tested classified into seven subgroups. Interestingly, the seven proteins from green alga were grouped into the same clade (Group 7), while those from land plants were grouped into the remaining clades (Group 1– Group 6).

As expected, SBP-domain proteins from grape generally exhibited closer relationships to SBP-like proteins from dicotyledonous angiosperms than to those from monocotyledonous angiosperms. These results suggest that while plant SBP-box genes may be derived from a common ancestor, a number of them may have undergone further differentiation in monocotyledon and dicotyledon lineages. Interestingly, the *miR156/157*-targeted SBP-like genes, including sequences from rice, *Arabidopsis* and grape, were distributed into only three of the subgroups (Groups 2, 3 and 4).

### *VvmiR156* Target Sites and Gene Structure of Grape SBP Genes

**Figure 3:**
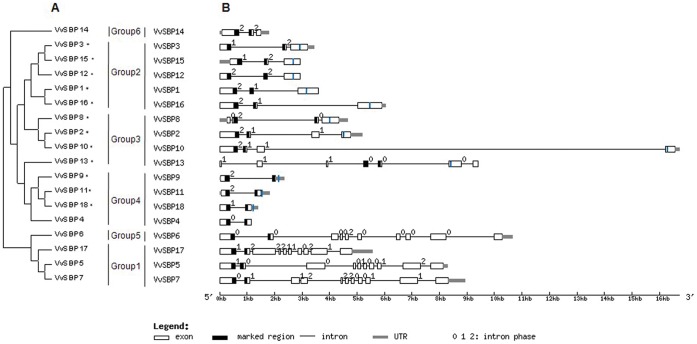
Genomic organization of SBP loci in grape. (A) Phylogenetic analysis of grape SBP-domain proteins. SBP-box genes that contained complementary sequences for *miR156/157* are marked with an asterisk. (B) Exon-intron structures of grape SBP genes. Untranslated5’ and 3′ regions, exons and SBP-domains are indicated by gray, white and black boxes, respectively. Black lines connecting two exons represent introns. The *miR156/157* target sites are denoted by blue vertical lines.

To compare the 18 grape SBP genes directly, a phylogenetic tree was constructed based on the full-length cDNAs (Fig. 3A) and their exon-intron structures were predicted (Fig. 3B). In general, the topology of the resulting phylogenetic tree was similar to that constructed with the 128 SBP-domain sequences from nine plant species (Fig. 2). In addition, the majority of grape SBP genes in the same group bore a similar number of exons, as well as length of open reading frames (ORFs). For example, all genes within Group 1 were made up of more than ten exons and 800 corresponding amino acid residues. Conversely, genes in Group4 all contained two exons and fewer than 210 corresponding amino acid residues, while genes in Groups2 and 3 had three and four exons, respectively, with the exception of *VvSBP13*, which contained seven exons. Interestingly, *VvSBP6* exhibited differences in the two phylogenetic analyses, which was the sole sequence classified into Group 5 in the multi-species tree (Fig. 2) but was clustered into Group 1 in the *V. vinifera*-specific tree (Fig. 3). However, it is noteworthy that the exon-intron structure of this gene was similar to that of other members of Group 1.

Furthermore, both the locations of *miR156/157* target sites and composition of encoded SBP domains were compared in each of the grape SBP genes to gain further insight into their evolutionary relationship with one another. Of the twelve grape SBP genes containing a *miR156/157* target site, Group 4 members (with the exception of *VvSBP4*) bore this site within their 3′ UTRs (Fig. 3B), which is similar to *AtSPL3*, *AtSPL4* and *AtSPL*5 in *Arabidopsis*. In addition, grape SBP proteins also contained a highly conserved SBP-domain bearing two zinc-binding sites (zinc finger 1 and zinc finger 2) and a nuclear localization signal (NLS). All SBP-domain proteins in Groups 1–4, as well as Group 6, possessed a zinc finger 1 (CR4CR13HCR2H) and zinc finger2 (CR2CR3HR11CR6H) of the C2HCH type, while *VvSBP6* contained a zinc finger 1 (CR4CR13HCR2C) of the C2HC2 type, as was also the case for other SBP-domain proteins within Group 5 (Fig. S2).

### Expansion Patterns and Distribution of Grape SBP and *miR156* Genes in the Grape Genome

**Figure 4:**
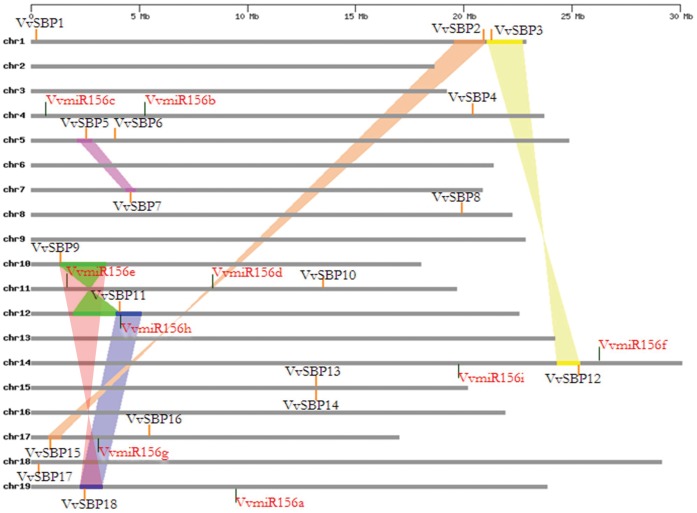
Chromosomal distribution of SBP and miR156 genes, as well as synteny of *SBP*-box genes in grape. *V. vinifera* chromosomes 1–19 (chr1–19) are depicted by horizontal gray bars. SBP and miR156 genes are indicated by vertical orange and black lines, respectively. Colored bars denote syntenic regions of the grape genome; twisted colored bars indicate that the syntenic regions are in reverse orientations.

According to available annotation information, the 18 grape SBP genes were dispersed on all grape chromosomes except for chromosomes 2, 3, 6, 9, 13 and 16. Three grape SBP genes were present on chromosome 1, two on chromosomes 5, 15 and 17, respectively, and one on each of the remaining chromosomes. In addition, the nine *VvmiR156* genes were found to be distributed on 6 of the 19 grape chromosomes: two were present on chromosomes 4, 11 and 14, respectively, and one was present on chromosomes 12, 17 and 19, respectively. Interestingly, as was the case for the grape SBP genes, none of the *VvmiR156/157* genes were present on chromosomes 2, 3, 6, 9, 13 or 16 (Fig. 4).

Moreover, we also examined duplicated blocks within the grape genome and found that nine grape SBP genes (*VvSBP2*/*VvSBP15*, *VvSBP3*/*VvSBP12*, *VvSBP5*/*VvSBP7*, and *VvSBP9*/*VvSBP11*/*VvSBP18*) were located in six pairs of duplicated genomic regions (Fig. 4, Table S4). In addition, according to their chromosomal positions, the*VvSBP13*/*VvSBP14* genes were present as a single tandem duplication.

### Evolutionary Relationship between SBP-box Family Genes of Grape and *Arabidopsis*

**Figure 5:**
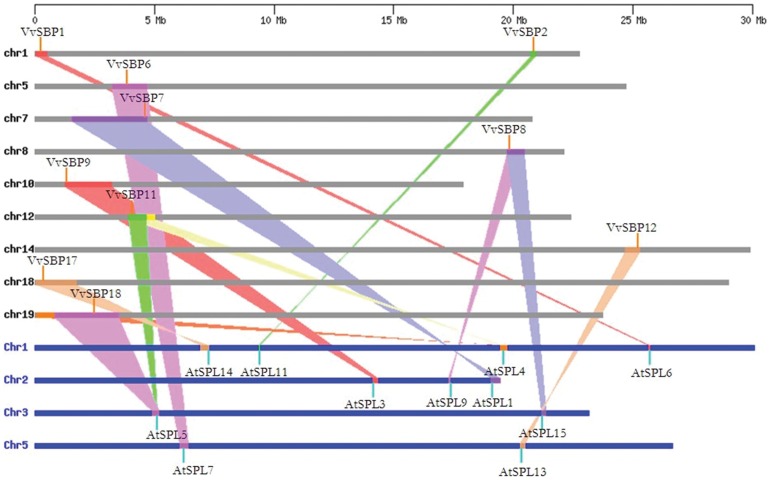
Synteny analysis of *SBP*-box genes of grape and *Arabidopsis*. *V. vinifera* and *Arabidopsis* chromosomes are depicted as horizontal gray and blue bars, respectively. Grape and *Arabidopsis* SBP-box genes are indicated by vertical orange and blue lines, respectively. Colored bars denote syntenic regions between grape and *Arabidopsis* chromosomes; a twisted colored bar indicates that the syntenic regions are in opposite orientations.

Genomic comparison is a relatively rapid method to transfer genomic knowledge acquired in one taxon to another that has been less well-studied. However, the degree to which genome synteny can facilitate cross-species analyses of gene function depends upon both the conservation of gene order and content, as well as the frequency with which similar traits have a common genetic basis in different species [25]. Since *Arabidopsis* is an important model plant species and the functions of most *Arabidopsis* SBP*-*box genes have been well-characterized, we analyzed a comparative synteny map between grape and *Arabidopsis* genomes in order to provide further insight into the functions of grape SBP-box genes. With regards to grape to *Arabidopsis* SBP-box gene correspondences, the syntenies were unambiguous and included the following ortholog pairs: *VvSBP1*-*AtSPL6*; *VvSBP2*-*AtSPL11*; *VvSBP6*-*AtSPL7*; *VvSBP7*-*AtSPL1*; *VvSBP8*-*AtSPL9,15*; *VvSBP9*-*AtSPL3*; *VvSBP11*,*18*-*AtSPL4,5*; *VvSBP12*-*AtSPL13*; and *VvSBP17*-*AtSPL14* (Fig. 5, Table S4).

### Expression Profiles of Grape SBP*-*box Genes in Different Organs and Stages of Fruit Development

**Figure 6:**
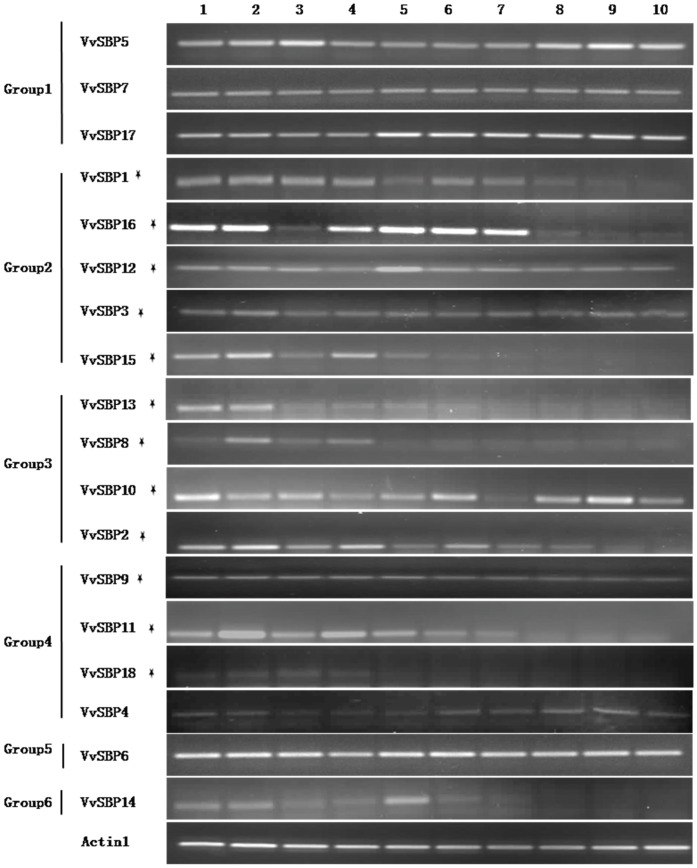
Tissue-specific expression pattern of grape SBP genes. Expression of grape SBP genes was analyzed in various organs and in fruits at different developmental stages from *V. quinquangularis* ‘Shang-24’ by semi-quantitative RT-PCR. *Actin1* was used as an internal control. Lane 1: leaves, 2: stems, 3: inflorescence, 4: tendrils, 5: fruit 20 daf, 6: fruit 35 daf, 7: fruit 50 daf, 8: fruit 65 daf, 9: fruit 80 daf, 10: fruit 95 daf.

To increase our understanding of the function of *SBP-*box genes in grape development, we investigated their expression profiles in various organs and at different stages of fruit development in *V. quinquagularis* ‘Shang-24’ via semi-quantitative RT-PCR with transcript-specific primers (Table S5). In general, the expression patterns of the 18 SBP-box genes could be classified into two types according to the presence or lack, of a *miR156/157* target site (Fig. 6). In the case of genes lacking a *miR156/157* target site, including *VvSBP4*, *VvSBP5*, *VvSBP6*, *VvSBP7*, *VvSBP14* and *VvSBP17*, there tended to be little or no variation in expression in any of the tissues tested. In contrast, genes containing a *miR156/157* target site, including*VvSBP1*, *VvSBP2*, *VvSBP3*, *VvSBP8*, *VvSBP9*, *VvSBP10*, *VvSBP11*, *VvSBP12*, *VvSBP13*, *VvSBP15*, *VvSBP16* and *VvSBP18*, were expressed at relatively higher levels in leaves, stems and tendrils compared to the reproductive tissues analyzed. Furthermore, the majority of these 12 grape SBP-box genes with *miR156/157* target sites also exhibited the highest levels of expression in the early stages of fruit development, which gradually decreased or even vanished during the fruit ripening process.

### Expression Profiles of Grape SBP-box Genes in Response to Various Types of Stresses

**Figure 7:**
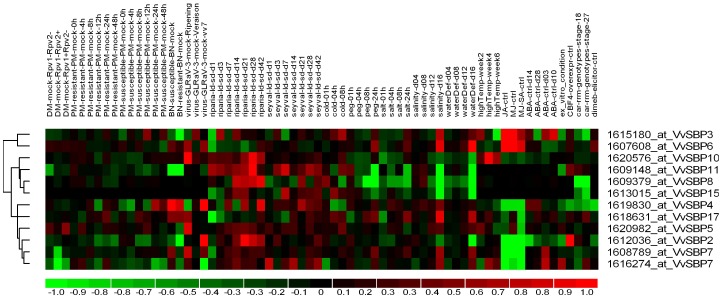
Hierarchical clustering of grape SBP-box genes. Details of the experimental conditions utilized are provided in Table S6. Log2-based fold changes were used to create the heat map. Differences in gene expression are shown in color according to the scale.

Plants undergo continuous exposure to various biotic and abiotic stresses in their natural environment. To improve the ability of a plant to tolerate stresses and improve disease resistances, it is necessary to identify and functionally characterize the genes that are involved in biotic and abiotic stress signaling pathways. Unfortunately, previous studies of SBP-box genes have focused mainly on plant growth and development, with only a handful having been shown to play a role in stress responses. Therefore, in the present study, we investigated the responses of grape SBP-box genes to various abiotic and biotic stress conditions, as well as hormone treatments, by mining publicly available grape microarray datasets. A total of 16 experiments containing 359 hybridizations from grape genome arrays were obtained and subjected to manual curation, and 68 comparisons between numerous distinct experimental conditions were constructed. We identified 11 grape SBP-box transcripts corresponding to 12 probe sets on the array, including *VvSBP2*, *VvSBP3*, *VvSBP4*, *VvSBP5*, *VvSBP6*, *VvSBP7*, *VvSBP8*, *VvSBP10*, *VvSBP11*, *VvSBP15* and *VvSBP17*. A heat map representation of the expression profiles of these genes is shown in Figure 7, which reveals that several of the grape SBP-box genes were highly responsive to each of the different stresses applied individually.

#### Abiotic stress

Abiotic stresses such as drought, extreme temperatures, and salinity can cause extensive losses to agricultural production worldwide. In the present study, we found that among the grape SBP-box genes affected by abiotic stress, six (*VvSBP3*, *VvSBP4*, *VvSBP5*, *VvSBP6*, *VvSBP7* and *VvSBP17*) were up-regulated by long-term exposure to salinity (4–16 days) while three (*VvSBP8*, *VvSBP11* and *VvSBP15*) were down-regulated (Table S6). Conversely, only two genes (*VvSBP7*and *VvSBP17*) were up-regulated by short-term salinity (1–24 h) while two (*VvSBP5* and *VvSBP11*) were down-regulated. Four genes (*VvSBP5*, *VvSBP6*, *VvSBP7* and *VvSBP17*) exhibited enhanced expression following water-deficit treatment (4–16 days), whereas four genes (*VvSBP2*, *VvSBP8*, *VvSBP10* and *VvSBP15*) were down-regulated. Furthermore, three genes (*VvSBP6*, *VvSBP7* and *VvSBP17*) were up-regulated following short-term PEG treatment (1–24 h), whereas two genes (*VvSBP8* and *VvSBP11*) were down-regulated (Table S6).

Following cold treatment (5°C), two genes (*VvSBP3* and *VvSBP5*) demonstrated increased expression, while two (*VvSBP4* and *VvSBP7*) exhibited decreased expression (Table S6). In contrast, following heat stress, we found that none of the grape SBP*-*box genes displayed any significant changes in expression levels (Table S6).

**Figure 8:**
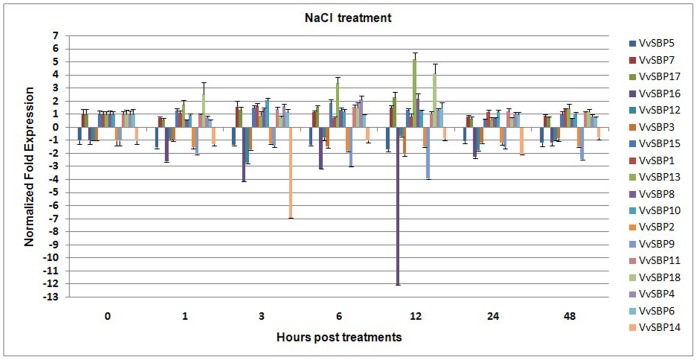
Expression levels of grape SBP-box genes following short-term salinity treatment in the leaves of *V. quinquangularis* ‘Shang-24’. Grape *Actin1* was used as an internal control for qRT-PCR and fold changes were used to indicate expression levels in treated leaves compared to negative controls, which were set to 1 or −1 depending on the expression trends. Mean values and SDs were obtained from three technical and three biological replicates.

To further investigate the responses of the grape SBP-box genes to abiotic stresses, we performed quantitative real-time RT-PCR (qRT-PCR) assays to test the expression of all 18 SBP-box genes in the leaves of Chinese wild *V. quinquangularis* clone ‘Shang-24’ upon short-term salinity (1–48 h) treatment (Fig. 8). Genes with expression levels altered by more than two-fold were considered for subsequent analyses. The results obtained showed that two genes (*VvSBP13* and *VvSBP18*) were strongly induced (over four-fold), and four genes (*VvSBP4*, *VvSBP8*, *VvSBP10* and *VvSBP17*) were also moderately up-regulated (over two-fold) in response to short-term salinity stress. In contrast, three genes (*VvSBP9*, *VvSBP14* and *VvSBP16*) were significantly down-regulated (over four-fold) and two genes (*VvSBP3* and *VvSBP12*) were moderately down-regulated (over two-fold) by this same treatment. The results obtained with qRT-PCR here were largely consistent with the array results.

#### Developmental and environmental cues

Photoperiod influences several aspects of plant growth, and is an important environmental cue for synchronizing plant growth, flowering, and dormancy with changing seasons [26]. Intriguingly, we found that all 11 grape SBP-box genes on the array were induced during long photoperiods in both *V. riparia* and *V.* spp. ‘Seyval’, indicating that the expression of SBP-box genes could be regulated by photoperiod (Table S6).

#### Biotic stress

Diverse diseases, such as downy mildew, powdery mildew, Bois Noir phytoplasma, and those of viral origin, have a serious impact on grapevine productivity and fruit quality. *Plasmopara viticola* is the causal agent of downy mildew, which is one of the most catastrophic and baffling diseases of grapevine worldwide [27]. Our microarray data mining revealed that only *VvSBP*7 exhibited a decreased expression upon *P. viticola* infection in a line that was highly resistant to this disease (Rpv1−/Rpv2+). Conversely, in both partially resistant (Rpv1+/Rpv2−) and susceptible (Rpv1−/Rpv2−) lines, none of the SBP-box genes displayed significant alterations in expression levels (Table S6).

Similarly, no significant changes in the expression levels of any of the 11 grape SBP-box genes analyzed were observed upon infection with powdery mildew in either the disease-resistant *V. aestivalis* ‘Norton’ or the disease-susceptible *V. vinifera* ‘Cabernet sauvignon’. In contrast, the expression of *VvSBP3* was significantly decreased after infection with Bois Noir phytoplasma in the grape cultivar ‘Manzoni’, which is moderately resistant to this disease, while in the highly susceptible ‘Chardonnay’, both *VvSBP4* and *VvSBP17* were significantly up-regulated and one gene (*VvSBP11*) was down-regulated. Lastly, the expression of *VvSBP5*, *VvSBP6*, *VvSBP7*, *VvSBP10* and *VvSBP17* were all up-regulated, while *VvSBP4* was down-regulated, in ripening berries of *V. vinifera* cv. ‘Cabernet Sauvignon’ when infected with leaf roll-associated closeterovirus-3 (GLRaV-3), which is one of the most widespread viruses among the more than 40 different viruses known to infect grapevines [28]. However, none of the grape SBP-box genes showed any significant changes in expression in response to GLRaV-3 at veraison (Table S6).

#### Hormone treatment

Plant hormones such as salicylic acid (SA), jasmonates (JA), ethylene (ET) and abscisic acid (ABA) play important roles in regulating developmental processes and signaling networks involved in plant responses to a wide range of biotic and abiotic stresses [29]. Analysis of expression data from red-skinned ‘Crimson Seedless’ grape (*V. vinifera*) cell-suspension cultures [30] indicated that the majority of grape SBP-box genes analyzed here were differentially expressed upon both JA and methyljasmonate (MeJA) treatment, with the exception of *VvSBP8*, *VvSBP11* and *VvSBP15*. Among the JA-responsive genes, two (*VvSBP3* and *VvSBP6*) were up-regulated, while six (*VvSBP2*, *VvSBP4*, *VvSBP5*, *VvSBP7*, *VvSBP10* and *VvSBP17*) were down-regulated. In the skin of grape berries treated with exogenous ABA [31], which is known to play a central role in the response of plants to various types of abiotic stresses, four of the 11 SBP genes analyzed exhibited altered levels of expression. Among them, three (*VvSBP4*, *VvSBP3* and *VvSBP7*) were up-regulated, while one (*VvSBP6*) was down-regulated (Table S6).

**Figure 9:**
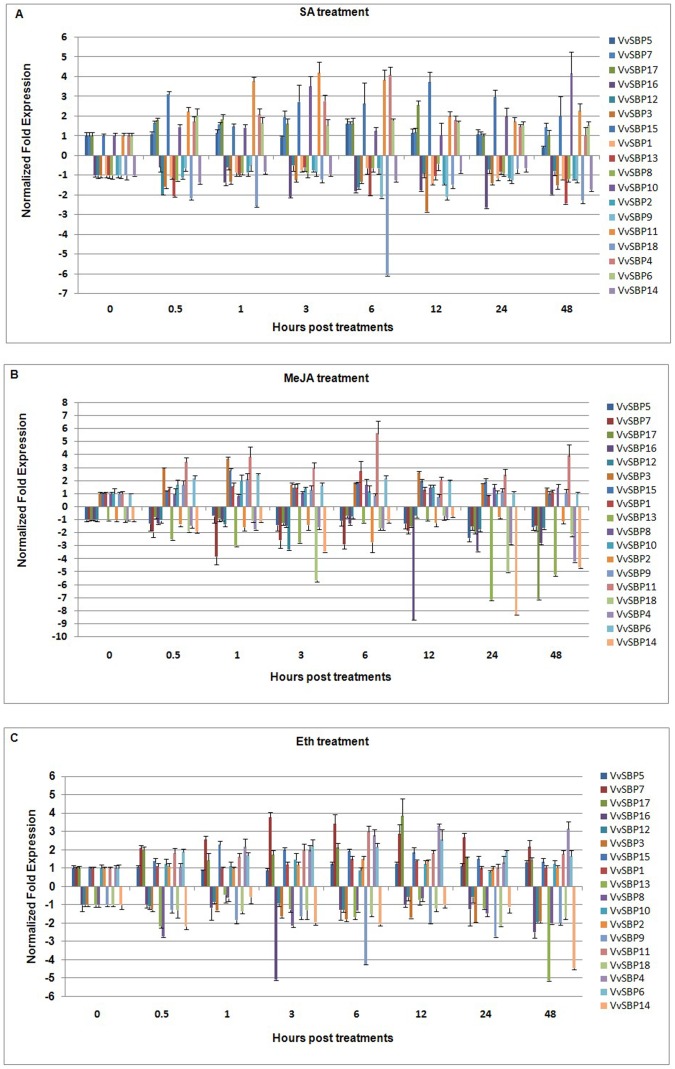
Expression levels of grape SBP-box genes following SA(A), MeJA(B), and ET (C) treatment in the leaves of *V. quinquangularis* ‘Shang-24’. Grape *Actin1* was used as an internal control for qRT-PCR and fold changes were used to indicate expression levels in treated leaves compared to negative controls, which were set to 1 or −1 depending on the expression trends. Mean values and SDs were obtained from three technical and three biological replicates.

To provide further insight into the responses of the grape SBP-box genes to signaling molecules, we carried out qRT-PCR assays to test the expression of all 18 SBP-box genes in the leaves of Chinese wild *V. quinquangularis* clone ‘Shang-24’ upon MeJA, ethylene (ET) and SA treatments (Fig. 9). Genes with expression levels altered by more than two-fold were considered for subsequent analyses.

Six hours following MeJA treatment, five genes (*VvSBP1*, *VvSBP6*, *VvSBP9*, *VvSBP11* and *VvSBP15*) exhibited significant increases in expression, while eight genes (*VvSBP2, VvSBP3, VvSBP4, VvSBP7*, *VvSBP13*, *VvSBP14, VvSBP16*, *VvSBP17* and *VvSBP18*) displayed significant decreases in their expression levels (Fig. 9B). The results obtained were consistent with the array results in that *VvSBP2, VvSBP3, VvSBP4, VvSBP7* and *VvSBP17* were significantly down-regulated by MeJA, and *VvSBP6* was moderately up-regulated by this same hormone, while the expression of the remaining three genes (*VvSBP5, VvSBP8* and *VvSBP10*) did not appear to be obviously altered by MeJA treatment.

With the exception of six genes (*VvSBP1*, *VvSBP2*, *VvSBP5*, *VvSBP7*, *VvSBP8* and *VvSBP14*) that did not exhibit any significant alterations in expression levels in response to SA, the expression levels of the remaining 12 grape SBP-box genes were significantly modified by this hormone (more than a two-fold increase or decrease). Of these, six genes (*VvSBP4*, *VvSBP6*, *VvSBP10*, *VvSBP11*, *VvSBP15* and *VvSBP17*) were up-regulated and six (*VvSBP*3, *VvSBP9*, *VvSBP12*, *VvSBP13*, *VvSBP16* and *VvSBP18*) were down-regulated (Fig. 9A).

Following treatment with ET, we found that six genes (*VvSBP4*, *VvSBP6*, *VvSBP7*, *VvSBP11*, *VvSBP15* and *VvSBP17*) were up-regulated and six genes (*VvSBP8*, *VvSBP9*, *VvSBP13*, *VvSBP14*, *VvSBP16* and *VvSBP18*) were down-regulated (Fig. 9C).

## Discussion

### Gene Duplication and Functional Diversification of Grape *SBP*-box Genes

It has been proposed that SBP-box genes encode a family of plant-specific transcription factors [1] as no SBP-box homologs have been identified in other kingdoms to date. Phylogenetic analyses demonstrated that all SBP-box genes from land plants were clustered into six groups (Groups 1–6), while the seven SBP-like genes identified from green alga fell into their own separate group (Group7, Fig. 2), which was consistent with previous findings [5]. These results imply that SBP-box genes arose after the divergence of plants and animals, but before the divergence of green algae from the last common ancestor of land plants.

Duplication at both the gene and genomic levels is one of the primary driving forces of genomic variation and gene family expansion [32], [33], contributing to the origin of biological novelty during evolution [34]. Gene duplications in angiosperms have been reported in many transcription factor families, such as the AP2, MADS, and DOF families [35]–[37], and two tandem SBP-box gene duplications have been reported in *Arabidopsis* (*AtSPL13A/AtSPL13B* and *AtSPL10/AtSPL11*) [38]. In the present study, we also identified a tandem duplication in the grape SBP genes (*VvSBP13*/*VvSBP14*, Fig. 4). Moreover, several segmental duplications of SBP-box gene pairs in *Arabidopsis* (*AtSPL10*/*AtSPL11*, *AtSPL4/AtSPL5*, and *AtSPL1*/*AtSPL12*) and rice (*OsSBP10*/*OsSBP5*, *OsSBP11*/*OsSBP4*, and *OsSBP12*/*OsSBP3*) have been identified [39]–[42]. Similarly, an examination of duplicated blocks within the grape genome indicated that nine of the grape SBP-box genes (*VvSBP2*/*VvSBP15*, *VvSBP3*/*VvSBP12*, *VvSBP5*/*VvSBP7*, and *VvSBP9*/*VvSBP11*/*VvSBP18*) were located in six pairs of duplicated genomic regions (Fig. 4, Table S4). Taken together, our results demonstrate that both segmental and tandem duplications have played important roles in the expansion of the grape SBP-box gene family during their evolution.

Comparative genomic analysis across different taxa allows the transfer of functional information from a taxon for which there is a better understanding of genome structure, function and/or evolution to another less well-studied taxon [43]. In this study, we found that the majority of grape and *Arabidopsis* SBP-box genes were located in syntenic regions of the two genomes (Fig. 5, Table S4). To date, several important and divergent biological processes regulated by SBP-box genes have been reported in *Arabidopsis*, including sporogenesis [6], leaf development [44], vegetative and reproductive phase transitions [4], response to copper and fungal toxins [15], [45] and plant hormone signaling [14]. Together with our expression data, these results will help infer the probable functions of grape SBP-box genes.

### Expression Profiles of Grape SBP-box Genes and their Potential Functions in Diverse Grape Tissues and Developmental Stages

SBP-box genes have been identified in numerous plants including green algae, moss, silver birch, snapdragon, *Arabidopsis*, rice, maize and tomato. To date, these genes have been found to play critical roles in regulating flower and fruit development, as well as various other physiological processes; however, their roles in grapevine have remained unclear. Therefore, in this study, we predicted the functions of the grape SBP-box genes in both vegetative and reproductive growth phases based on their *Arabidopsis* counterparts in syntenic regions of the two genomes (Fig. 5) and homology to genes of other species.

In general, semi-quantitative RT-PCR results demonstrated that the grape SBP-box genes exhibited distinct expression patterns. However, four pairs of grape SBP-box genes (*VvSBP2*/*VvSBP15*, *VvSBP3*/*VvSBP12*, *VvSBP5*/*VvSBP7*, and *VvSBP11*/*VvSBP18*) sharing very high sequence and exon–intron structure similarity in duplicated genomic regions (Fig. 3), also showed similar expression patterns (Fig. 6). Although *VvSBP11* exhibited a higher level of expression than *VvSBP18* in most grape tissues and organs, both of these Group 4 genes had elevated expression levels in inflorescences and during the early stages of fruit development, which then gradually decreased during veraison (Fig. 6). These results suggest that these two genes may play a role in flower and fruit development. This assumption is corroborated by the fact that the *Arabidopsis AtSPL5* gene, which is the ortholog of *VvSBP11*/*VvSBP18*, was reported to be involved in the flowering process. In addition, the snapdragon gene *AmSBP1*, as well as the *Arabidopsis* genes *AtSPL3* and *AtSPL4*, were also clustered into Group 4 in our phylogenetic analysis. Intriguingly, *AmSBP1* and*AtSPL3* have also been reported to bind *cis*-elements in the promoters of the floral organ identity genes *SQUA* and *AP1*, respectively [2], and *AtSPL3*/*4*/*5*, as well as *AmSBP1,* have all been implicated in the vegetative phase change and floral induction [2], [4], [10].

Interestingly, grape*VvSBP2* and *VvSBP15*, which are located in duplicated genomic regions, were not classified into the same subgroup in our phylogenetic analysis (Fig. 2). However, they did exhibit similar expression patterns in the various grape developmental phases tested. For example, the expression of these two genes was elevated in vegetative organs (leaves, stems and tendrils), and gradually diminished throughout the fruit maturation process (Fig. 6). It is worth noting that the expression of *VvSBP15* and *VvSBP2* waned completely in fruits at 50 and 80 days after flowering, respectively. These findings suggest that these two genes may play a regulatory role in leaf initiation and during different stages of grape fruit ripening. In addition, grape *VvSBP8* and *VvSBP13*, which were distributed into the same group as *VvSBP2* (Group 3), both shared similar trends during the fruit maturation process. Therefore, we speculate that the Group 3 grape SBP-box genes might function in both vegetative and reproductive stages, a hypothesis that is further supported by previous studies. For instance, *AtSPL9* and *AtSPL15*, which are orthologs of grape *VvSBP8*, are quite active in the vegetative shoot apex and play a role in the juvenile-to-adult phase transition [11]. In addition, other members of Group 3 have also been implicated in both vegetative and reproductive processes. Indeed, *AtSPL10/11/2* are involved in the development of lateral organs, shape of cauline leaves and number of trichomes on cauline leaves and flowers [46], while *OsSPL14* promotes panicle branching in the vegetative stage and increased rice crop yield in the reproductive stage [47]. In addition, *ZmSBP6* (*tasselsheath4*) has been found to be involved in inflorescence development in maize while an SBP-box gene from tomato (*CNR*) plays a role in fruit ripening [18], [19].

In contrast, the Group 2 grape genes *VvSBP3* and *VvSBP12* were expressed in all organs analyzed and grape members of this group were homologous to *AtSPL13*, *teosinte glume architecture 1* (*tga1*) and *OsSPL6*. The *Arabidopsis AtSPL13* gene has been shown to affect the initiation of the first true leaves [44], maize *tga1*is involved in ear glume development [48], and *OsSPL16* controls grain size, shape and quality in rice [49]. Therefore, we hypothesize that the grape Group 2 SBP-box genes may provide functions in controlling characteristics of leaf and/or fruit.

The three groups of SBP-box genes discussed above (Groups 2, 3, and 4), with the exception of *SBP4*, all contain a *miR156/157* target site. This miRNA family is highly conserved in plants, with homologs having been identified in a large number of angiosperms, ferns, lycopods and mosses [50]–[52]. Previously, nine members of the *miR156/157* family, termed *VvmiR156a* to *VvmiR156i*, which are highly conserved in plants and are thought to interact with numerous SBP-box genes, were identified in the *V. vinifera* genome [53]–[56]. To date, *miR156/157* target sites were found in 10 *Arabidopsis* [57], 11 rice [21] and 10 tomato [23] SBP-box genes. Indeed, 12 of 18 grape SBP-box genes contained a *miR156/157* target site in the *V. vinifera* genome (Fig. 1). In most cases, *miR156/157*-regulated SBP-box genes tend to play a role in the control of phase change and reproductive development [58], [59]. It has been shown that the grape *VvmiR156f* gene is expressed at the highest levels in mature berries, followed by inflorescences, ripening berries, green berries and leaves [53]. Our experimental results, which were consistent with the expression patterns of *miR156/157*-targeted genes in tomato and rice [21], [23], indicated that the *miR156/157*-targeted grape SBP-box genes were generally expressed in a similar fashion to the *VvmiR156f* gene in all tissues tested (Fig. 6). This provides yet another example of the mutual relationship between *miR156/157* and SBP-box genes.

In contrast to the grape SBP-box genes discussed above, Group 1 and Group 5 genes did not contain a *miR156/157* target site and were all expressed ubiquitously and constitutively, with little or no variation in any of the tissues analyzed (Fig. 6). These results indicate that grape genes from these two groups may have functions that are distinct from the *miR156/157*-targeted SBP-box genes in Groups 2, 3 and 4.

### Grape *SBP-*box Genes are Responsive to Abiotic and Biotic Stresses

Transcriptional control of stress-responsive genes is a crucial means by which plants respond to a range of abiotic and biotic stresses and research carried out in recent years has been productive in identifying transcription factors that are important for regulating these types of responses [60]. To date, several important and divergent biological processes regulated by SBP-box genes have been reported; however, only a small number of these genes have been shown to play a role in the response to stresses. For example, in *Arabidopsis*, the expression of an SBP-box gene has been found to be responsive to various types of biotic and abiotic stresses through interactions with genes involved in the defense response pathway [61]. In addition, *AtSPL14* has been found to be involved in programmed cell death and plays a role in sensitivity to fumonisin B1 [45]. In grape, we found that *VvSBP17*, which is the orthologof *AtSPL14*, was up-regulated after infection with Bois Noir in the susceptible *V. vinifera* cultivar ‘Chardonnay’. Furthermore, grape *VvSBP7* exhibited down-regulated expression upon *P. viticola* infection in a highly resistant line. Similarly, *VvSBP17*, *VvSBP7* and *VvSBP5* all exhibited significant increases in expression levels following infection with GLRaV-3 in ripening berries. Intriguingly, these three genes all belonged to the same subgroup (Group 1), which insinuates that the Group 1 grape SBP-box genes may play a key role in the response to pathogen infection in grape. Moreover, our microarray data mining revealed that both *VvSBP4* and *VvSBP11* from Group 4, as well as *VvSBP3* from Group 2, also displayed significant alterations in expression levels after infection with pathogens (Fig. 7, Table S6).

Among the diverse grape SBP-domain proteins, the SBP domain of VvSBP6 showed the highest similarity to that of AtSPL7 from *Arabidopsis*, which regulates Cu deficiency responses [15]. Moreover, the SBP-box gene, *Cu response regulator 1* (*Crr1*), from *C. reinhardtii* fulfills a very similar role [20]. Therefore, it is possible that *VvSBP6*, which is the most likely *AtSPL7* ortholog and single grape representative in Group 5, is also an important regulator of copper homeostasis in this species. In addition, *VvSBP6* also exhibited responsiveness to abiotic stress, being up-regulated by drought, PEG treatment and salinity (Fig. 7, Table S6).

The existence of multiple defense strategies and complex signaling networks in plants has led to their enhanced defense capacity. Induced defense responses are regulated by a network of interconnected signal transduction pathways in which the hormonal signals SA, JA, ET and ABA, can coordinately activate the transcription of various defense-related genes [62]. In this sense, these plant hormones provide a critical function in signaling networks involved in plant responses to a wide range of biotic and abiotic stresses. To date, the only report of an SBP-box gene exhibiting responsiveness to biotic stress signaling hormones has come from *AtSPL*2, which was found to be repressed in transgenic *Arabidopsis* overexpressing the *JASMONATE CARBOXYL METHYLTRANSFERASE* gene (*AtJMT*) [63]. In this study, we determined that the expression of the majority of grape SBP-box genes was significantly modified by SA, MeJA and/or ET treatment (Fig. 8, Table S6). It is worth noting that grape SBP genes responsive to pathogen or virus infection (*VvSBP3*, *VvSBP4*, *VvSBP5*, *VvSBP6*, *VvSBP7*, *VvSBP10*, *VvSBP11* and *VvSBP17*) were also responsive to at least one of the three hormone treatments. SA, JA and ET have been reported to be involved in the defense not only during pathogen infection but also during salt stress. The ameliorative effects of SA to salt tolerance have been well documented in many crops such as bean [64], mung bean [65] and mustard [66]. JA can also activate plant defense mechanisms and provide protection to salinity stress [67]–[71]. A recent study suggests that ethylene signaling may be required for salt tolerance and promote salt tolerance in *Arabidopsis* [72], [73]. This study suggests that grape SBP genes may be involved in the response to salt stress dependent on MeJA and ET molecular signals. However, their roles in salt stress are equivocal. Furthermore, ABA is extensively involved in responses to abiotic stress, such as water-deficit, cold and osmotic stress [74], and also acts as a negative regulator of disease resistance in many cases [75]. In our study, five of the 11 grape SBP-box genes analyzed were also found to be regulated by ABA (Fig. 7, Table S6). Taken together, our analyses indicate that SBP-box genes have a variety of functions in grape and play important roles in both biotic and abiotic stresses, possibly dependent on SA, MeJA, ET and ABA molecular signals.

## Materials and Methods

### Identification and Annotation of Grape SBP-box Genes

To identify members of the SBP-box gene family in grape, previously identified *Arabidopsis SBP* sequences were first submitted to the Pfam database (http://pfam.sanger.ac.uk) [76] to obtain the domain architecture of this family. SBP motifs were found to be represented by Pfam accession number PF03110. Searches for each domain within the Grape Genome Database (12X; http://www.genoscope.cns.fr) were performed using HMMER [77] with an E-value of <1e^−5^. To confirm results obtained using the HMMER algorithm, protein motifs were also queried against the Pfam database.

### Sequence Alignments and Phylogenetic Analyses

Multiple alignments of SBP-domain protein sequences derived from 128 SBP-box genes from 9 plant species (Table S2) were performed using the ClustalW program [78]. Phylogenetic trees were constructed using the MEGA 4.0 software and the maximum parsimony (MP) method, with a bootstrap test that was replicated 1000 times [79].

### Exon/Intron Structure Analysis of Grape SBP-box Genes

The exon/intron structures of the grape SBP genes were determined from alignments of their coding sequences with corresponding genomic sequences using the est2genome program [80]. A diagram of exon/intron structures was obtained using the online Gene Structure Display Server (GSDS: http://gsds.cbi.pku.edu.ch), which depicts both exon position and gene length.

### Tandem Duplication and Synteny Analysis

Tandem duplications of SBP genes in the *V. vinifera* genome were predicted by determining their physical locations on individual chromosomes. Tandemly duplicated genes were defined as adjacent homologous genes on a single chromosome, with no more than one intervening gene. For synteny analysis, syntenic blocks within the *V. vinifera* genome, as well as between the grape and *Arabidopsis* genomes, were downloaded from the Plant Genome Duplication Database [81] and those containing grape and *Arabipidopsis* SBP-box genes were identified.

### Expression Analysis of Grape SBP-box Genes

Affymetrix grape microarray data were downloaded from ArrayExpress [82] and PLEXdb [83] databases. A total of 16 experiments were used for our gene expression analyses (Table S6). For each microarray experiment, the methods utilized for normalization and to adjust background, as well as detection calls, P-value calculation and adjustment have been described previously [84]. Genes with adjusted p-values (FDR) less than 0.05 were considered to be differentially expressed genes. Hierarchical clustering of the expression profiles of the grape SBP-box genes was performed using dChip [85].

### Plant Material and Treatments

Grape tissues, including leaf, stem, tendril, inflorescence, and fruit at 20, 35, 50, 65, 80 and 95 days after flowering (daf), were obtained from *V. quinquangularis* ‘Shang-24’, which had been maintained in the grape germplasm resource orchard of Northwest A&F University, Yangling, China (34°20′, 108°24′E). Grape seedlings were grown in pots under greenhouse conditions. When shoots of vines were 25–35 cm in length, the third to fifth fully expanded young grapevine leaves beneath the apex were selected for SA, MeJA and ET treatments. Hormone treatments were conducted by spraying leaves with 100 µM SA, 0.5 g/L ET or 50 µM MeJA followed by sampling at 0, 0.5, 1, 3, 6, 12, 24 and 48 h post-treatment as described previously [86]. Grape leaves sprayed with sterile water were collected as the control. Salinity treatments were carried out by irrigating with 2 dm^3^250 mMNaCl. Grapevine leaves were sampled at 0, 1, 3, 6, 12, 24 and 48 h post-treatment. Leaves of grape irrigating with water were collected as the control.

### Isolation of Complete Open Reading Frames of Grape SBP Genes

For each SBP gene, a pair of gene-specific primers (Table S1) were designed to amplify the predicted ORF with cDNA templates prepared from leaves of *V. quinquangularis* ‘Shang-24’. *Taq* DNA polymerase (TaKaRa Biotechnology, Dalian, China) was used to amplify the grape SBP genes with the following cycling profile: 94°C for 3 min, 25 to 30 cycles at 94°C for 30 s, 58°C for 30 s and 72°C for 2 or 3 min; and extension at 72°C for 10 min. The amplified products were cloned into pGEM-Teasy vector (Promega, Madison, WI, USA) and transformed into *E.coli* strain DH5α. The positive clones, characterized by blue/white screening, were sequenced at TaKaRa Biotechnology.

### Quantitative Real-time RT-PCR Analysis

Total RNA was extracted from the leaves of *V. quinquangularis* ‘Shang-24’ using an improved SDS/phenol method described previously [87]. Residual DNA was removed via treatment with DNaseI (Promega, Madison, WI, USA). RNA purity was ascertained by determining the A_260_/A_280_ ratio, and RNA integrity was examined through electrophoresis on a 1% agarose gel. Concentrations of total RNA were measured using an ultraviolet spectrophotometer (V-550, JASCO, Japan) at 260 nm. First-strand cDNA synthesis was carried out using 1 µg DNase-treated total RNA and a mixture of Poly dT and random hexamer primers (PrimeScript™ RTase, TaKaRa Biotechnology, Dalian, China). Gene-specific primers were designed for all 18 grape SBP-box genes (Table S5). Quantitative RT-PCR was conducted using SYBR green (Takara Biotechnology) on an IQ5 real time PCR machine (Bio-Rad, Hercules, CA, USA). Each reaction was done in triplicates with a volume of 25 µl. Cycling parameters were 95°C for 30 s, 40 cycles of 95°C for 5 s, and 60°C for 30 s. For dissociation curve analysis, a program including 95°C for 15 s, followed by a constant increase from 60°C to 95°C, was included after the PCR cycles. The grape *Actin1* (GenBank Accession number AY680701) was amplified with primers F (5′-GAT TCT GGT GAT GGT GTG AGT-3′) and R (5′-GAC AAT TTC CCG TTC AGC AGT-3′) as an internal control. Relative expression levels were analyzed using the IQ5 software and the normalized-expression method. A one-sided paired *t*-test was performed using Sigma Plot 11.0 (Ashburn, VA, USA) to assess significant differences between the control and each treatment.

### Semi-quantitative RT-PCR Analysis

Total RNAs were extracted from leaf, stem, tendril, inflorescence, and fruit at 20, 35, 50, 65, 80 and 95 daf of *V. quinquangularis* ‘Shang-24’ as described above. Grape *SBP* and *Actin1* specific primer sequences were the same as those used for qRT-PCR. Cycling parameters were as follows: 94°C for 3 min, 25 cycles at 94°C for 30 s, 60°C for 30 s and 72°C for 30 s, with a final elongation step at 72°C for 10 min. PCR products were subsequently separated on a 1.2% (w/v) agarose gel, then stained with ethidium bromide and photographed under UV light.

## Supporting Information

Figure S1 **Agarose gel electrophoresis test of 18 grape SBP-box genes.**
(TIF)

Figure S2 **Sequence logos (A) and multiple alignment (B) of the SBP domain in grape; multiple alignment (C) and sequence logos (D) of the SBP domains from Group 5 proteins of all plant species analyzed.**
(TIF)

Table S1 **Primers for grape SBP genes ORF amplification.**
(DOC)

Table S2 **Data sources of selected SBP-box genes for phylogenetic analysis.**
(DOC)

Table S3 **SBP-domain sequences and accession numbers of selected plant SBP-box genes used for phylogenetic analysis.**
(DOC)

Table S4 **Syntenic blocks between grape and** ***Arabidopsis*** **SBP genes.**
(XLS)

Table S5 **Grape SBP-box gene-specific primer sequences for RT-PCR and sequencing reactions.**
(DOC)

Table S6 **Details of publicly available grape array datasets and grape SBP expression profiles.**
(XLS)
