# Supplementary figures and images for: Genomic Organization, Phylogenetic Comparison and Differential Expression of the SBP-Box Family Genes in Grape
Source: PLoS One. 2013 Mar 19;8(3):e59358. doi: 10.1371/journal.pone.0059358 (PMC3601960; doi:10.1371/journal.pone.0059358)

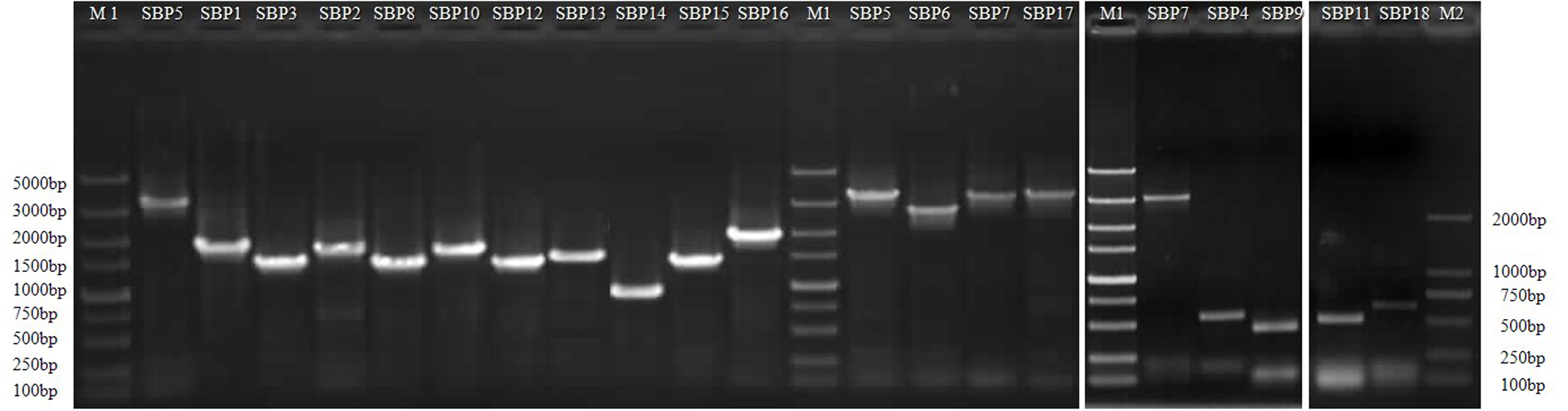

Supplement: Figure S1 — Agarose gel electrophoresis test of 18 grape SBP-box genes. (TIF) [file pone.0059358.s001.tif]

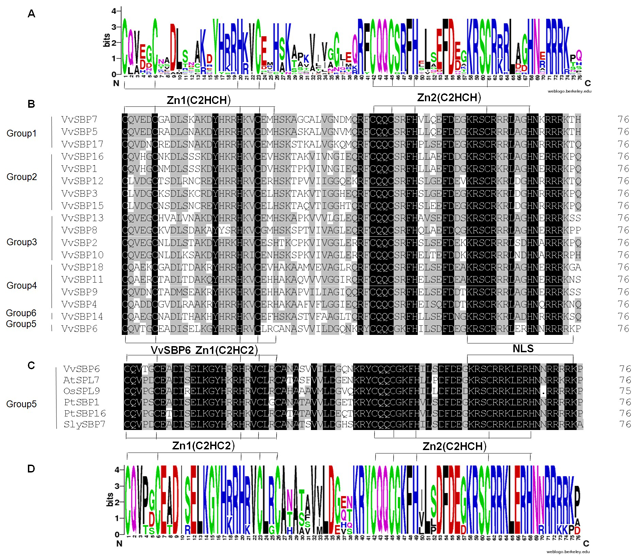

Supplement: Figure S2 — Sequence logos (A) and multiple alignment (B) of the SBP domain in grape; multiple alignment (C) and sequence logos (D) of the SBP domains from Group 5 proteins of all plant species analyzed. (TIF) [file pone.0059358.s002.tif]
