# Supplementary material for: Genomic Organization, Phylogenetic Comparison and Differential Expression of the SBP-Box Family Genes in Grape
Source: PLoS One. 2013 Mar 19;8(3):e59358. doi: 10.1371/journal.pone.0059358 (PMC3601960; doi:10.1371/journal.pone.0059358)
Supplement: Table S1 — Primers for grape SBP genes ORF amplification. (DOC) [file pone.0059358.s003.doc]

Table S1. Primers for grape SBP genes ORF amplification

| Gene | Accession No. or locus ID | Chrom | Forward and Reverse primers(5’-3’) | CDS (bp) | ORF (aa) | Counterparts in  Wang et al [24] |
| --- | --- | --- | --- | --- | --- | --- |
| *VvSBP1* | XM_002273498.1  (GSVIVT01012247001) | 1 | f: ATGGATTTAACAACTGATGC  R: TTAAGTAGTAGGAAAACAG | 1524 | 507 | *VvSBP15* |
| *VvSBP*2 | XM_002271276.1  (GSVIVT01010496001) | 1 | f:ATGATTTCTTTCTCAATGATG  R: TCAGTTCAACTGACCAG | 1434 | 477 |  |
| *VvSBP*3 | XM_002270190  (GSVIVT01010522001) | 1 | f: ATGGGCTATAATCTGAAGAC  R: CTACTCCCAAGAGAACG | 1170 | 389 | *VvSBP*9 |
| *VvSBP4* | XM_002274219.1  (GSVIVT01003836001) | 4 | f: ATGGCGAAGAGATCGTTAGA  R: tcacctaattcgaaaatgcc | 624 | 206 | *VvSBP11* |
| *VvSBP5* | XM_002274898  (GSVIVT01017678001) | 5 | f: ATGGAGCACAAATTCGGA  R: TTAGCTGGATCCATACTTCA | 3090 | 1029 | *VvSBP8* |
| *VvSBP6* | XM_002277003.1  (GSVIVT01017835001) | 5 | f: ATGGAGACATCATCACTTCC  R: CTAAGAATTGTCAAAC | 2406 | 801 | *VvSBP3* |
| *VvSBP*7 | XM_002273192.1  (GSVIVT01028208001) | 7 | f: ATGGAGGCTAAAATTGGGG  R: TCAGCTTGTGCCATAGTCCA | 2994 | 997 | *VvSBP*16 |
| *VvSBP8* | XM_002278476.1  (GSVIVT01033519001) | 8 | f: ATGGAAAGGGGTTCGAG  R: CTAAAGTGACCAGTGC | 1137 | 378 |  |
| *VvSBP9* | XM_002280016.1  (GSVIVT01021087001) | 10 | f: ATGGAAATGAGCAAGGCTCA  R: TCAGCTGGAGCCTTCTCCAT | 423 | 140 | *VvSBP7* |
| *VvSBP10* | XM_002267188.1  (GSVIVT01032239001) | 11 | f: ATGGAGTGGAATTTAAGAA  R: TCAGTTAATTTGATTGGGA | 1398 | 465 |  |
| *VvSBP*11 | XM_002275692.1  (GSVIVT01020578001) | 12 | f: ATGGAAGCTAAGAAGATGGT  R: TCATCTGATCTGGAAATGC | 510 | 169 | *VvSBP*4 |
| *VvSBP12* | XM_002274324.1  (GSVIVT01033064001) | 14 | f: ATGGAGTGGAACTTACAGCC  R:TCATGAGGGCTCATTTTCTGA | 1137 | 378 | *VvSBP12* |
| *VvSBP13* | XM_002274466.1  (GSVIVT01018205001) | 15 | f: ATGGTGAACACATCAAATG  R: TCATACCGTACCTTCCTTCG | 1230 | 409 | *VvSBP5* |
| *VvSBP*14 | XM_002277147.1  (GSVIVT01018204001) | 15 | f: ATGCTGGACTACGAATG  R: TCACCCACTCGGGAAAAAC | 915 | 304 | *VvSBP*19 |
| *VvSBP15* | XM_002280124.1  (GSVIVT01008556001) | 17 | f: ATGGACTGGAACTTGAAGAC  R: CTAATCCCAGAAGAAGGGAA | 1134 | 377 | *VvSBP6* |
| *VvSBP*16 | XM_002265167.1 | 17 | f: ATGGGGTCTTGGAGCTA  R: TTACGTTATCCGGAGGC | 1674 | 557 | *VvSBP*1 |
| *VvSBP*17 | XM_002273748.1  (GSVIVT01013452001) | 18 | f: ATGGAAGAGGTTGGTG  R: CTAACTTGTACCATAAT | 3213 | 1070 | *VvSBP*13 |
| *VvSBP*18 | XM_002282562.1  (GSVIVT01014302001) | 19 | f: ATGGAATCCAAGTCTTCTAC  R: TCATCTGATCTGGAAATGC | 615 | 204 | *VvSBP*17 |
