# Supplementary material for: Genomic Organization, Phylogenetic Comparison and Differential Expression of the SBP-Box Family Genes in Grape
Source: PLoS One. 2013 Mar 19;8(3):e59358. doi: 10.1371/journal.pone.0059358 (PMC3601960; doi:10.1371/journal.pone.0059358)
Supplement: Table S2 — Data sources of selected SBP-box genes for phylogenetic analysis. (DOC) [file pone.0059358.s004.doc]

Table S2. Data sources of selected SBP-box genes for phylogenetic analysis

| Lineage | Organism | Number | Nomenclature | Data sources |
| --- | --- | --- | --- | --- |
| Alga | *Chlamydomonas reinhardtii* | 7 | CRR1 or CrSPL | (Guo et al., 2008) |
| Moss | *Physcomitrella patens* | 13 | PpSBP | (Guo et al., 2008) |
| Dicots | *Arabidopsis thaliana* | 16 | AtSPL | http://datf.cbi.pku.edu.cn |
|  | *Populus trichocarpa* | 26 | PtSBP | [http://dptf.cbi.pku.edu.cn](http://dptf.cbi.pku.edu.cn/) |
|  | *Vitis vinifera* | 18 | VvSBP | http://www.genoscope.cns.fr and GenBank |
|  | *Solanum lycopersicum* | 15 | CNR or SlySBP | (Salinas et al., 2011) |
|  | *Antirrhinum* *majus* | 5 | AmSBP or AmQ9SNV | （Xie et al., 2006） |
| Monocots | *Oryza sativa* | 19 | OsSBP | <http://rice.tigr.org/> |
|  | *Zea mays* | 9 | ZmSBP | <http://maize.TIGR.org/> |
| Total |  | 128 |  |  |
