# Supplementary material for: Genomic Organization, Phylogenetic Comparison and Differential Expression of the SBP-Box Family Genes in Grape
Source: PLoS One. 2013 Mar 19;8(3):e59358. doi: 10.1371/journal.pone.0059358 (PMC3601960; doi:10.1371/journal.pone.0059358)
Supplement: Table S3 — SBP-domain sequences and accession numbers of selected plant SBP-box genes used for phylogenetic analysis. (DOC) [file pone.0059358.s005.doc]

Table S3. SBP-domain sequences and accession numbers of selected plant SBP-box genes used for phylogenetic analysis

| Gene name | Protein sequence of SBP-domain | Accession No. or locus ID |
| --- | --- | --- |
| VvSBP1 | CQVHGCNMDLSSSKDYHKRHKVCDVHSKTPKVIVNGIEQRFCQQCSRFHLLAEFDDGKRSCRKRLAGHNERRRKPQ | XM_002273498.1(GSVIVT01012247001) |
| VvSBP2 | CQVEGCNLDLSTAKDYHRKHRVCESHTKCPKVIVGGLERRFCQQCSRFHSLSEFDEKKRSCRRRLSDHNARRRKPQ | XM_002271276.1(GSVIVT01010496001) |
| VvSBP3 | CLVDGCKSDLSKCRDYHRRHKVCELHSKTAKVTIGGHEQRFCQQCSRFHSLGEFDEGKRSCRKRLDGHNRRRRKPQ | XM_002270190(GSVIVT01010522001) |
| VvSBP4 | CQADDCGVDLRAAKRYHRRHKVCERHAKAAFVFLGGIEQRFCQQCSRFHEISQFDDTKRSCRKRLAGHNQRRRKNQ | XM_002274219.1(GSVIVT01003836001) |
| VvSBP5 | CQVEDCRADLGNAKDYHRRHKVCDMHSKASKALVGNVMQRFCQQCSRFHLLQEFDEGKRSCRRRLAGHNRRRRKTH | XM_002274898(GSVIVT01017678001) |
| VvSBP6 | CQVTGCEADISELKGYHRRHRVCLRCANASVVILDGQNKRYCQQCGKFHILSDFDEGKRSCRRKLERHNNRRRRKP | XM_002277003.1(GSVIVT01017835001) |
| VvSBP7 | CQVEDCGADLSKAKDYHRRHKVCEMHSKAGCALVGNDMQRFCQQCSRFHVLQEFDEGKRSCRRRLAGHNKRRRKTH | XM_002273192.1(GSVIVT01028208001) |
| VvSBP8 | CQVEGCKVDLSDAKAYYSRHKVCGMHSKSPTVIVAGLEQRFCQQCSRFHQLAEFDQGKRSCRRRLAGHNERRRKPP | XM_002278476.1(GSVIVT01033519001) |
| VvSBP9 | CQVDNCTADMSEAKRYHKRHRVCEHHAKAPVILIAGIQQRFCQQCSRFHELSEFDDTKRSCRRRLAGHNERRRKSS | XM_002280016.1(GSVIVT01021087001) |
| VvSBP10 | CQVEGCNLDLKSAKDYHRRHRICENHSKSPKVIVAGLERRFCQQCSRFHELTEFDDKKRSCRRRLNDHNARRRRPH | XM_002267188.1(GSVIVT01032239001) |
| VvSBP11 | CQAERCTADLTDAKQYHRRHKVCEHHAKAQVVVVGGIRQRFCQQCSRFHELSEFDEAKRSCRRRLAGHNERRRKNS | XM_002275692.1(GSVIVT01020578001) |
| VvSBP12 | CLVDGCTSDLRNCREYHRRHRVCERHSKTPVVIIGGQEKRFCQQCSRFHSLGEFDEVKRSCRKRLDGHNRRRRKTQ | XM_002274324.1(GSVIVT01033064001) |
| VvSBP13 | CQVEGCHVALVNAKDYHRRHKVCEMHSKAPKVVVLGLEQRFCQQCSRFHAVSEFDDSKRSCRRRLAGHNERRRKSS | XM_002274466.1(GSVIVT01018205001) |
| VvSBP14 | CQAEGCNADLTHAKHYHRRHKVCEFHSKASTVFAAGLTQRFCQQCSRFHLLSEFDNGKRSCRKRLADHNRRRRKSQ | XM_002277147.1(GSVIVT01018204001) |
| VvSBP15 | CLVDGCNSDLSNCREYHRRHKVCELHSKTAQVTIGGHTQRFCQQCSRFHSLEEFDEGKRSCRKRLDGHNRRRRKPQ | XM_002280124.1(GSVIVT01008556001) |
| VvSBP16 | CQVHGCNKDLSSSKDYHKRHKVCEVHSKTAKVIVNGIEQRFCQQCSRFHLLAEFDDGKRSCRKRLAGHNERRRKPQ | XM_002265167.1 |
| VvSBP17 | CQVDNCREDLSNAKDYHRRHKVCEMHSKSTKALVGKQMQRFCQQCSRFHPLSEFDEGKRSCRRRLAGHNRRRRKTQ | XM_002273748.1(GSVIVT01013452001) |
| VvSBP18 | CQAEKCGADLTDAKRYHRRHKVCEVHAKAAMVEVAGLRQRFCQQCSRFHELSEFDEAKRSCRRRLAGHNERRRKGA | XM_002282562.1(GSVIVT01014302001) |
| AmSBP1 | CQVENCAAEMTNAKPYHRRHKVCEFHAKAPVVLHSGLQQRFCQQCSRFHELSEFDEAKRSCRRRLAGHNERRRKSS | Q38741 |
| AmSBP2 | CLVENCGADLRNCKKYYQRHRVCEVHAKAPVVSVEGLMQRFCQQCSRFHDLSEFDQTKRSCRRRLAGHNERRRKSS | Q38740 |
| AmQ9SNV3 | CQVEGCEVDLSDAKAYYLRHKVCSMHSKSPKVIVAGIEQRFCQQCSRFHQLPEFDQGKRSCRRRLAGHNERRRKPS | Q9SNV3 |
| AmQ9SNV4 | CQVEGCKIDLSDAKAYYSRHKVCGMHSKSPKVIVAGIEQRFCQQCSRFHQLPEFDQGKRSCRRRLAGHNERRRKPP | Q9SNV4 |
| AmQ9SNV5 | CQAEGCNADLSHCKHYHRRHKVCEFHSKAANVVAAGLTQRFCQQCSRFHVLSEFDNGKRSCRRRLADHNRRRRKSS | Q9SNV5 |
| CRR1 | CQVPGCGKDLTNLKEYHQRYRICDVHIKLQQVLKDGRLQRFCQQCGRFHDLTAFDGNRKSCRDQLSKHNARRRRRA | 148821 (AY484394) |
| CrSPL2 | CQVDKCNQAMACQQEQYQRLRICEHHYHALEVQHEGMRQRFCQSCGRLHPVEEFESNMHACRARCGVDSRQRKRLR | 93505 |
| CrSPL3 | CQVEGCGHDLTGEKGYYQRYRICEPHVKLLTVEVEGRACRFCQQCGRFHELSEFDGTKRSCRARLLLHNARRRKRD | 186869 |
| CrSPL4 | CHVQGCNRSLAGLRDYYQRYKICEHHLKVSQVLKDGVPHRFCQQCGRFHPLTEFDGDRRSCRTMLQRHCHRRAKQK | 191464 |
| CrSPL5 | CHVEGCTVDLASAGPYYQRYRTCELHLKAPYIIKDGMQQRFCQQCGRFHELSEFDNNKRSCRARWGHHNQRRRKRT | 101247 |
| CrSPL6 | CHVDDCNVDLSSLKEYHQRFRICDFHLKAEVVLREGIPQRFCQQCGRFHLLSEFDGTKRSCRARWVCGGSTHRSST | 101657 |
| CrSPL7 | CQVGGCTADLTDVKKYFQRYRICERHLKAPSLKMDGRAVRFCDQCSKFHDIGAFDGARRTCTEKLEKTRQARVALN | 171833 |
| ZmSBP1 | CQAEGCKADLSAAKHYHRRHKVCEYHAKASAVAAAGKQQRFCQQCSRFHVLAEFDEAKRSCRKRLTEHNRRRRKPS | AJ011614 |
| ZmSBP2 | CQAEGCKADLSAAKHYHRRHKVCEFHTKASAVAAAGKQQRFCQQCSRFHVLAEFDEAKRSCRKRLTEHNRRRRRKP | AJ011615 |
| ZmSBP3 | CQAEGCKADLSGAKHYHRRHKVCEYHAKASVVATGGKQQRFCQQCSRFHVLSEFDEVKRSCRKRLAEHNRRRRKPP | AJ011616 |
| ZmSBP4 | CQAEGCKADLSGAKHYHRRHKVCEYHAKASVVAANGKQQRFCQQCSRFHVLSEFDEVKRSCRKRLAEHNRRRRKPA | AJ011617 |
| ZmSBP5 | CQVDGCNVDLTDVKAYYCRHKVCKMHSKEPRVLVNGLEQRFCQQCSRFHQLPEFDQLKKSCRKRLAGHNERRRRPP | AJ011618 |
| ZmSBP6 | GVDLSAVKQYYCRHKVCNMHSKEPRVFVAGIEQRFCQQCSRFHQLHEFDQGKRSCRRRLIGHNERRRKPP | AJ011619 |
| ZmSBP7 | CLVDGCNADLTDAKTYYCRHKVCEMHSKEPRVVVNGLELRFCQQCSRFHNLAEFDQQKKSCRKRLAGHNERRRRP- | AJ011620 |
| ZmSBP8 | CQAEGCKADLSSAKRYHRRHKVCEHHSKAPVVVTAGLHQRFCQQCSRFHLLDEFDDAKKSCRKRLADHNRRRRKSK | U89496 |
| ZmSBP9 | CQVEGCHLSLADAKEYHRRHKVCEAHSKAPRVVVLGAEQRFCQQCSRFHAISEFDDAKRSCRRRLAGHNERRRKSN | CO517866 |
| PpSBP1 | CQAEGCKDDLSNAKHYHRRHKVCELHSKAPTVTVGGHTQRFCQQCSRFHHLGEFDEGKRSCRKRLADHNRRRRKPQ | AJ968320 |
| PpSBP2 | CQVDGCTADLSRAKDYHRRHKVCEAHSKAPTTLVSRVRQRFCQQCSRFHPLDKFDEDKRSCRRRLAGHNKRRRKTQ | AJ968403 |
| PpSBP3 | CQVQGCDADLSCCKDYHKRHKVCEMHSKAATAIAAGIEQRFCQQCSRFHVLKEFDEGKRSCRRRLAGHNQRRRKSQ | AJ968318 |
| PpSBP4 | CQAEGCKTDLSTSKQYHRRHKVCELHSKAPNVQVGGQTQRFCQQCSRFHSLEEFDNGKRSCRKRLADHNRRRRKPQ | AJ968319 |
| PpSBP5 | CQVPACGADLAGLKGYHQRHRVCLQCAKAPKCIAAGIEQRFCQQCSRFHVLTEFDEGKRSCRRRLAGHNERRRKPH | EF016491 |
| PpSBP6 | CQVEGCKADLSGCKDYHKRHKVCEMHSKAPKCIAAGIEQRFCQQCSRFHVLTEFDEGKRSCRRRLAGHNERRRKPH | EF016492 |
| PpSBP7 | CQAEGCKSDLSTAKQYHRRHKVCELHSKAPNVVAGGQTQRFCQQCSRFHSLGEFDDGKRSCRKRLADHNRRRRKPQ | EF016493 |
| PpSBP8 | CQAEGCKFDLSLAKPYHRRHKVCELHSKAPNVIAGGQTQRFCQQCSRFHSLGEFDDGKRSCRKRLADHNRRRRKPQ | fgenesh1_pg.194000034 |
| PpSBP9 | CQAEGCKADLNVTKNYYRRHKVCEFHSKTPIVIVGGHTQRFCQQCSRFHRLGEFDDGKRSCRKRLADHNRRRRKAQ | EF016494 |
| PpSBP10 | CQVDGCTADLSKAKDYHRRHKVCETHSKASTAQVSRVTQRFCQQCSRFHALDQFDEGKRSCRRRLAGHNKRRRKTQ | EF016495 |
| PpSBP11 | CQVDACKADLSKAKDYYRRHKVCETHSKATKAPVSRLMQRFCQQCSRFHPLQEFDEGKRSCRRRLAGHNRRRRKTQ | EF647594 |
| PpSBP12 | CQAKGCKADLSLAKQYHRRHKVCEHHSKALNVVANGQTQRFCLQCSRFHLLGEFDDGKRTCPKRLANHNRRRRKPQ | EF016496 |
| PpSBP13 | CQVEGCKADLSGCKDYHKRHKVCEMHSKAPKCIAAGIEQRFCQQCSRFHVLTEFDEGKRSCRRRLAGHNERRRKPH | EF016497 |
| PtSBP1 | CQVPSCEADISELKGYHRRHRVCLGCANATAVVLDGETKRYCQQCGKFHVLSDFDEGKRSCRRKLERHNNRRRRKP | estExt_fgenesh4_pm.C_LG_X0096 |
| PtSBP2 | CQVEDCRADLSNAKDYHRRHKVCDVHSKASMALVGNVMQRFCQQCSRFHVLQEFDEGKRSCRRRLAGHNKRRRKTH | gw1.X.791.1 |
| PtSBP3 | CQVKNCTTDMTDAKRYHKRHKVCEFHAKASSVLVNGVEQRFCQQCSRFHDLSEFDDSKRSCRRRLAGHNERRRKSS | gw1.IV.3037.1 |
| PtSBP4 | CQADNCTSDLADAKRYHRRHKVCEFHAKAPFAPVNGLQQRFCQQCSRFHDLSEFDDSKRSCRRRLAGHNERRRKSS | estExt_Genewise1_v1.C_1240186 |
| PtSBP5 | CQVEKCGANLTDAKRYHRRHKVCEVHAKSPAVVVAGLRQRFCQQCSRFHELVEFDETKRSCRRRLAGHNERRRKST | gw1.107.39.1 |
| PtSBP6 | CQVEKCGANLTDAKRYHRRHKVCEVHAKSPAVVVAGLRQRFCQQCSRFHELLEFDETKRSCRRRLAGHNERRRKNT | gw1.XI.3794.1 |
| PtSBP7 | CQVEDCGVDLSNAKDYHRRHKVCEMHSKASKALVGNVMQRFCQQCSRFHVLQEFDEGKRSCRRRLAGHNKRRRKTN | gw1.8978.5.1 |
| PtSBP8 | CQVEKCTANLTDAKQYHRRHKVCGHHAKAQVVLVAGIRQRFCQQCSRFHELSEFDETKRSCRRRLAGHNERRRKNV | gw1.VII.548.1 |
| PtSBP9 | CQAEGCNADLTHAKHYHRRHKVCEFHSKASTVIAAGLTQRFCQQCSRFHILSEFDNGKRSCRKRLADHNRRRRKS- | gw1.II.3778.1 |
| PtSBP10 | CQAEGCNADLTHAKHYHRRHKVCEFHSKASTVIAAGLTQRFCQQCSRFHLLSEFDNGKRSCRKRLADHNRRRRKS- | gw1.40.81.1 |
| PtSBP11 | CQVEGCHVALLNAKDYHRRHKVCEMHSKAPKVIVLGLEQRFCQQCSRFHVVSEFDDAKRSCRRRLAGHNERRRKSS | gw1.40.76.1 |
| PtSBP12 | CLVDGCTSDLSKCRDYHRRHKVCEFHSKSSQVFIKGQEQRFCQQCSRFHSLGEFDEGKRSCRKRLDGHNRRRRKPQ | gw1.I.7783.1 |
| PtSBP13 | CQVEGCKVDLSDAKTYYSRHKVCSMHSKSPRVIVAGLVQRFCQQCSRFHLLPEFDQGKRSCRRRLAGHNERRRKPP | eugene3.00160416 |
| PtSBP14 | CLVDGCNSDLSACRDYHRRHKVCELHSKTPQVTVGGQKQRFCQQCSRFHSLEEFDEGKRSCRKRLDGHNRRRRKPQ | estExt_Genewise1_v1.C_LG_XV2187 |
| PtSBP15 | CQVYDCNKDLSSSKEYHKRHKVCEVHSRTAKVIVNGIEQRFCQQCSRFHLLAEFDDGKRSCRKRLAGHNERRRK-- | gw1.129.152.1 |
| PtSBP16 | CQVPGCETDISELKGYHRRHKVCLRCATATAVVLDEQTKRYCQQCGKFHVLSDFDEGKRSCRRKLERHNNRRRRKP | estExt_fgenesh4_pm.C_LG_VIII0830 |
| PtSBP17 | CQVEDCGVDLSNAKDYHRRHKVCEMHSKASKALVGNAMQRFCQQCSRFHVLQEFDEGKRSCRRRLAGHNKRRRKTN | gw1.II.489.1 |
| PtSBP18 | CQVEGCHVALVNAKGYHRRHKVCEMHSKAAKVIVLGLEQRFCQQCSRFHVVSEFDDAKRSCRRRLAGHNERRRKGS | fgenesh4_pg.C_LG_II001303 |
| PtSBP19 | CQVYDCNKDLSSSKDYHKRHKVCEVHTKTPQVIVNGNEQRFCQQCSRFHLLVEFDDGKRSCRKRLAGHNERRRKPQ | fgenesh4_pg.C_LG_X001404 |
| PtSBP20 | CLVDGCNSDLSTCRDYHRRHKVCELHSKTPQVTIGGQKQRFCQQCSRFHSLEEFDEGKRSCRKRLDGHNRRRRKPQ | eugene3.00120942 |
| PtSBP21 | CQVEGCNLDLKSAKDYHRRHRICEKHSKSPKVIVAGMERRFCQQCSRFHELSEFDDKKRSCRRRLSDHNARRRR-- | gw1.164.76.1 |
| PtSBP22 | CQVEGCNLDLSSAKDYHRKHRVCESHSKCQKVIVAGLERRFCQQCSRFHGLSEFDEKKKSCRRRLSDHNARRRKQP | gw1.I.7690.1 |
| PtSBP23 | CLVDGCTSDLTKCRDYHRRHKVCELHSKSRQVFIKGQEQRFCQQCSRFHSLGEFDEGKRSCRKRLDGHNRRRRKS- | gw1.III.2396.1 |
| PtSBP24 | CQVDNCKENLTTAKDYHRRHKVCEVHSKATKALVGKQMQRFCQQCSRFHPLTEFDEGKRSCRRRLAGHNRRRRKTQ | eugene3.00051637 |
| PtSBP25 | CQVDNCKEDLSKAKDYHRRHKVCQVHSKATKALVGKQMQRFCQQCSRFHPLTEFDEGKRSCRRRLAGHNRRRRKTQ | fgenesh4_pm.C_LG_II000008 |
| PtSBP26 | CQVEDCGVDLSNAKDYHRRHKVCEMHSKASKALVGNAMQRFCQQCSRFHVLQEFDEGKRSCRRRLAGHNKRRRKTN | gw1.XIV.2149.1 |
| OsSPL1 | CQVDGCTVNLSSARDYNKRHKVCEVHTKSGVVRIKNVEHRFCQQCSRFHFLQEFDEGKKSCRSRLAQHNRRRRKVQ | LOC_Os01g18850.1 |
| OsSPL2 | CSVEGCAADLSKCRDYHRRHKVCEAHSKTAVVTVAGQQQRFCQQCSRFHLLGEFDEEKRSCRKRLDGHNKRRRKPQ | LOC_Os01g69830.1 |
| OsSPL3 | CQVEGCNVDLSSAKPYHRKHRVCEPHSKTLKVIVAGLERRFCQQCSRFHGLAEFDQKKRSCRRRLHDHNARRRKPQ | LOC_Os02g04680.1 |
| OsSPL4 | CQVEGCGVELVGVKDYHRKHRVCEAHSKFPRVVVAGQERRFCQQCSRFHALSEFDQKKRSCRRRLYDHNARRRKPQ | LOC_Os02g07780.1 |
| OsSPL5 | CQAEGCKADLSAAKHYHRRHKVCDFHAKAAAVLAAGKQQRFCQQCSRFHVLAEFDEAKRSCRKRLTEHNRRRRKPT | LOC_Os02g08070.1 |
| OsSPL6 | CQVEGCTADLTGVRDYHRRHKVCEMHAKATTAVVGNTVQRFCQQCSRFHPLQEFDEGKRSCRRRLAGHNRRRRKTR | LOC_Os03g61760.1 |
| OsSPL7 | CQVEGCDITLQGVKEYHRRHKVCEVHAKAPRVVVHGTEQRFCQQCSRFHVLAEFDDAKKSCRRRLAGHNERRRRSN | LOC_Os04g46580.1 |
| OsSPL8 | CQAEGCKADLSSAKRYHRRHKVCEHHSKAPVVVTAGLHQRFCQQCSRFHLLDEFDDAKKSCRKRLADHNRRRRKSK | LOC_Os04g56170.1 |
| OsSPL9 | CQVPGCEADIRELKGYHRRHRVCLRCAHAAAVMLDGVQKRYCQQCGKFHILLDFDEDKRSCRRKLERHNRRRRKPD | LOC_Os05g33810.1 |
| OsSPL10 | CQAEGCKADLSGAKHYHRRHKVCEYHAKASVVAASGKQQRFCQQCSRFHVLTEFDEAKRSCRKRLAEHNRRRRKPA | LOC_Os06g44860.1 |
| OsSPL11 | CQVEGCGLELGGYKEYYRKHRVCEPHTKCLRVVVAGQDRRFCQQCSRFHAPSEFDQEKRSCRRRLSDHNARRRKPQ | LOC_Os06g45310.1 |
| OsSPL12 | CQVEGCKVDLSSAREYHRKHKVCEAHSKAPKVIVSGLERRFCQQCSRFHGLAEFDQKKKSCRRRLSDHNARRRKPQ | LOC_Os06g49010.1 |
| OsSPL13 | CQVERCGVDLSEAGRYNRRHKVCQTHSKEPVVLVAGLRQRFCQQCSRFHELTEFDDAKRSCRRRLAGHNERRRKSA | LOC_Os07g32170.1 |
| OsSPL14 | CQVEGCGADLSGIKNYYCRHKVCFMHSKAPRVVVAGLEQRFCQQCSRFHLLPEFDQGKRSCRRRLAGHNERRRRPQ | LOC_Os08g39890.1 |
| OsSPL15 | CQVDDCRADLTNAKDYHRRHKVCEIHGKTTKALVGNQMQRFCQQCSRFHPLSEFDEGKRSCRRRLAGHNRRRRKTQ | LOC_Os08g40260.1 |
| OsSPL16 | CAVDGCKEDLSKCRDYHRRHKVCEAHSKTPLVVVSGREMRFCQQCSRFHLLQEFDEAKRSCRKRLDGHNRRRRKPQ | LOC_Os08g41940.1 |
| OsSPL17 | CQVEGCGVDLSGVKPYYCRHKVCYMHAKEPIVVVAGLEQRFCQQCSRFHQLPEFDQEKKSCRRRLAGHNERRRKPT | LOC_Os09g31438.1 |
| OsSPL18 | CAVDGCKADLSKHRDYHRRHKVCEPHSKTPVVVVSGREMRFCQQCSRFHLLGEFDEAKRSCRKRLDGHNRRRRKPQ | LOC_Os09g32944.1 |
| OsSPL19 | CSVDGCRSDLSRCRDYHRRHKVCEAHAKTPVVVVAGQEQRFCQQCSRFHNLAEFDDGKKSCRKRLDGHNRRRRKPQ | LOC_Os11g30370.1 |
| CNR | CQVDQCTADMADAKPYHRRHKVCEFHSKSPIVLISGLQKRFCQQCSRFHLLAEFDDAKRSCRRRLAGHNERRRKIT | Solyc02g077920 |
| SlySBP2 | CQVDSCNLDLSSAKQYHRKHRVCHIHSKCPKVIILGRHRRFCQQCSRFHSLSDFDENKRSCRTRLSDHNARRRKPQ | Solyc04g045560 |
| SlySBP3 | CQVEECTADMVNAKTYHRRHKVCEFHAKAPEVLIDGLRQRFCQQCSRFHQLAEFDDAKKSCRRRLAGHNERRRKSA | Solyc10g009080 |
| SlySBP4 | CQAEKCNVDLSDAKQYHKRHKVCEYHAKSQVVVVAGLRQRFCQQCSRFHELTEFDESKRSCRRRLAGHNERRRKST | Solyc07g053810 |
| SlySBP6a | CQVQGCGKDLSPCKDYHKRHKVCEVHSKTAKVIVNGIEQRFCQQCSRFHLLAEFDDGKRSCRKRLAGHNERRRKPP | Solyc03g114850 |
| SlySBP6b | CQVHGCNKDLSSSKDYHKRHKVCNEHSKTAIVIVNGIEQRFCQQCSRFHLLAEFDEGKRSCRKRLAGHNERRRKPQ | Solyc05g012040 |
| SlySBP6c | CQVQGCGKDLTSCKDYHKRHKVCEIHSKTAKVIVNGIQQRFCQQCSRFHLLAEFDDGKRSCRKRLAGHNERRRKPH | Solyc12g038520 |
| SlySBP7 | CQVPDCEADISELKGYHKRHRVCLRCANATSVVLDGHSKRYCQQCGKFHILSDFDEGKRSCRRKLERHNNRRRRKA | Solyc01g080670 |
| SlySBP8a | CQAEGCNADLTHAKHYHRRHKVCEFHSKASTVIAAGITQRFCQQCSRFHLLSEFDNGKRSCRKRLADHNRRRRKNQ | Solyc10g018780 |
| SlySBP8b | CQAEGCNADLTHAKHYHRRHKVCEFHSKASTVIAAGLTQRFCQQCSRFHVLSEFDNGKRSCRKRLADHNRRRRKNI | Solyc01g090730 |
| SlySBP10 | CQVEGCNLDLSSAKEYYRKHRVCDSHSKSPKVIVAGVARRFCQQCSRFHSVSEFDDKKRSCRRRLSDHNARRRKPH | Solyc05g015510 |
| SlySBP12a | CQVDDCGTDLSKAKDYHRRHKVCEMHSKASRALVGNVMQRFCQQCSRFHALQEFDEGKRSCRRRLAGHNKRRRKTQ | Solyc01g068100 |
| SlySBP12b | CQVQDCRADLSSAKDYHRRHKVCEVHSKAAKALVGNVMQRFCQQCSRFHVLEEFDEGKRSCRRRLAGHNKRRRKTH | Solyc05g053240 |
| SlySBP13 | CLVDGCNADLSECREYHRRHKVCEVHSKTAKVTIAGRDQRFCQQCSRFHSLVEFDDGKRSCRKRLDGHNRRRRKPQ | Solyc05g015840 |
| SlySBP15 | CQVEGCQADLSDAKAYYSRHKVCGMHSKSPTVVVAGLEQRFCQQCSRFHQLTEFDQGKRSCRRRLACHNERRRKPP | Solyc10g078700 |
| AtSPL1 | CQVENCEADLSKVKDYHRRHKVCEMHSKATSATVGGILQRFCQQCSRFHLLQEFDEGKRSCRRRLAGHNKRRRKTN | At2g47070 |
| AtSPL2 | CQVEGCNLDLSSAKDYHRKHRICENHSKFPKVVVSGVERRFCQQCSRFHCLSEFDEKKRSCRRRLSDHNARRRKPN | At5g43270 |
| AtSPL3 | CQVESCTADMSKAKQYHKRHKVCQFHAKAPHVRISGLHQRFCQQCSRFHALSEFDEAKRSCRRRLAGHNERRRKST | At2g33810 |
| AtSPL4 | CQVDRCTADMKEAKLYHRRHKVCEVHAKASSVFLSGLNQRFCQQCSRFHDLQEFDEAKRSCRRRLAGHNERRRKSS | At1g53160 |
| AtSPL5 | CQVDRCTVNLTEAKQYYRRHRVCEVHAKASAATVAGVRQRFCQQCSRFHELPEFDEAKRSCRRRLAGHNERRRKIS | At3g15270 |
| AtSPL6 | CQVYGCSKDLSSSKDYHKRHRVCEAHSKTSVVIVNGLEQRFCQQCSRFHFLSEFDDGKRSCRRRLAGHNERRRKPA | At1g69170 |
| AtSPL7 | CQVPDCEADISELKGYHKRHRVCLRCATASFVVLDGENKRYCQQCGKFHLLPDFDEGKRSCRRKLERHNNRRKRKP | At5g18830 |
| AtSPL8 | CQAEGCNADLSHAKHYHRRHKVCEFHSKASTVVAAGLSQRFCQQCSRFHLLSEFDNGKRSCRKRLADHNRRRRKCH | At1g02065 |
| AtSPL9 | CQVEGCGMDLTNAKGYYSRHRVCGVHSKTPKVTVAGIEQRFCQQCSRFHQLPEFDLEKRSCRRRLAGHNERRRKPQ | At2g42200 |
| AtSPL10 | CQIDGCELDLSSSKDYHRKHRVCETHSKCPKVVVSGLERRFCQQCSRFHAVSEFDEKKRSCRKRLSHHNARRRKPQ | At1g27370 |
| AtSPL11 | CQIDGCELDLSSAKGYHRKHKVCEKHSKCPKVSVSGLERRFCQQCSRFHAVSEFDEKKRSCRKRLSHHNARRRKPQ | At1g27360 |
| AtSPL12 | CQVDNCGADLSKVKDYHRRHKVCEIHSKATTALVGGIMQRFCQQCSRFHVLEEFDEGKRSCRRRLAGHNKRRRKAN | At3g60030 |
| AtSPL13 | CLVDGCDSDFSNCREYHKRHKVCDVHSKTPVVTINGHKQRFCQQCSRFHALEEFDEGKRSCRKRLDGHNRRRRKPQ | At5g50570 |
| AtSPL14 | CQVDNCTEDLSHAKDYHRRHKVCEVHSKATKALVGKQMQRFCQQCSRFHLLSEFDEGKRSCRRRLAGHNRRRRKTT | At1g20980 |
| AtSPL15 | CQVEGCRMDLSNVKAYYSRHKVCCIHSKSSKVIVSGLHQRFCQQCSRFHQLSEFDLEKRSCRRRLACHNERRRKPQ | At3g57920 |
| AtSPL17 | CLVDGCDSDFSNCREYHKRHKVCDVHSKTPVVTINGHKQRFCQQCSRFHALEEFDEGKRSCRKRLDGHNRRRRKPQ | At5g50670 |
