# Supplementary material for: Genomic Organization, Phylogenetic Comparison and Differential Expression of the SBP-Box Family Genes in Grape
Source: PLoS One. 2013 Mar 19;8(3):e59358. doi: 10.1371/journal.pone.0059358 (PMC3601960; doi:10.1371/journal.pone.0059358)
Supplement: Table S5 — Grape SBP-box gene-specific primer sequences for RT-PCR and sequencing reactions. (DOC) [file pone.0059358.s007.doc]

Table S5. Grape SBP-box gene-specific primer sequences for RT-PCR and sequencing reactions

| Gene | Forward primers(5’-3’) | Reverse primers(5’-3’) | Production length (bp) |
| --- | --- | --- | --- |
| *VvSBP1* | f:TCGGGGGAGAGGAGTCAAGTTCG | R:GCCGGCATGGATGAACCTAGCGA | 175 |
| *VvSBP*2 | f:TGCAGGGGGCAATGCTAAAGGCT | R: TCAACTTGGCAGCGCGAGACA | 117 |
| *VvSBP*3 | f:GGTTGGGTGATTTGGGGGACGGG | R:GCCCTCTTTGCTGACCCCGACG | 85 |
| *VvSBP4* | f:TTGCCAGCAGTGTAGCAGATTCCA | R:TTGTCGTCCGAATGCTCTGTGGC | 198 |
| *VvSBP5* | f:GCAGCACGCCAACATTGCAAGC | R:ACGCGTAAAGCACTTCCGGCGA | 169 |
| *VvSBP6* | f:acttcacagtagtgggggttt | R:ggcagcaatggcaagttcaac | 159 |
| *VvSBP*7 | f:TCTCAAGCTCCCCCACGGCCTATT | R:CTGCCCTGCAATACTGTCTCTCACT | 192 |
| *VvSBP8* | f:CGGCCAAATCGGGAACCGGG | R:TCTTTGGCGGCGCTGGAGGT | 81 |
| *VvSBP9* | f:TCATGCCAAGGCTCCGGTCATTCT | R:TCGTCAAACTCTGACAGCTCATGG | 93 |
| *VvSBP10* | f:AGCTGGTTTGGAGCGCCGGT | R:GGCAGCCTTCCAGAGCTGAATTGG | 162 |
| *VvSBP*11 | f:TCCGGCAGCGCTTCTGTCAGC | R:TCAGCTGAGTCCTTTCTGCGCCT | 124 |
| *VvSBP12* | f:AGACCATCAGCTCAGGCCCACA | R:GGTCGTTCGCCTTGGTGGTGTCG | 123 |
| *VvSBP13* | f:CGACGGTGCCGAGATGCCAGG | R:CCTGCAGCTCCTCTTTGAGTCGTCG | 200 |
| *VvSBP*14 | f:CAGAGCCAGCCTCAGCCCGC | R:GGCCACCGGCTCAAGCGACA | 105 |
| *VvSBP15* | f:CGCCGGCCATGGCTGCAAAAT | R:TGCAGGCTTGGGGCTGAGGG | 174 |
| *VvSBP*16 | f:GCCCAATTCGtCTGCAAGAAGT | R:CACCACCCTTGCCACATGAAACA | 200 |
| *VvSBP*17 | f:AATCGGAGCTGCTGCGCCTGG | R:AGCTCAAGCCCCCACCCAGC | 153 |
| *VvSBP*18 | f:gcagcgatggttgaggtggcc | R:TCCTCCGCCTCTCATTGTGGC | 136 |
